# The prognostic value of autophagy related genes with potential protective function in Ewing sarcoma

**DOI:** 10.1186/s12859-022-04849-x

**Published:** 2022-07-28

**Authors:** Jian Wen, Lijia Wan, Xieping Dong

**Affiliations:** 1 Medical College of Nanchang University, Nanchang, 330006 Jiangxi China; 2 Department of Orthopedics, Jiangxi Provincial People’s Hospital, 152 Aiguo Road, Nanchang, 330006 Jiangxi China; 3 JXHC Key Laboratory of Digital Orthopedics (Jiangxi Provincial People’s Hospital), 152 Aiguo Road, Nanchang, 330006 Jiangxi China; 4 Department of Pediatrics, The Second Xiangya Hospital, Central South University, Changsha, 410011 Hunan China

**Keywords:** Autophagy related genes, Protective, Ewing sarcoma, Survival, Prognostic model

## Abstract

**Background:**

Ewing sarcoma (ES) is the second most common primary malignant bone tumor mainly occurring in children, adolescents and young adults with high metastasis and mortality. Autophagy has been reported to be involved in the survival of ES, but the role remains unclear. Therefore, it’s necessary to investigate the prognostic value of autophagy related genes using bioinformatics methods.

**Results:**

*ATG2B*, *ATG10* and *DAPK1* were final screened genes for a prognostic model. KM and risk score plots showed patients in high score group had better prognoses both in training and validation sets. C-indexes of the model for training and validation sets were 0.68 and 0.71, respectively. Calibration analyses indicated the model had high prediction accuracy in training and validation sets. The AUC values of ROC for 1-, 3-, 5-year prediction were 0.65, 0.73 and 0.84 in training set, 0.88, 0.73 and 0.79 in validation set, which suggested high prediction accuracy of the model. Decision curve analyses showed that patients could benefit much from the model. Differential and functional analyses suggested that autophagy and apoptosis were upregulated in high risk score group.

**Conclusions:**

*ATG2B*, *ATG10* and *DAPK1* were autophagy related genes with potential protective function in ES. The prognostic model established by them exhibited excellent prediction accuracy and discriminatory capacities. They might be used as potential prognostic biomarkers and therapeutic targets in ES.

**Supplementary Information:**

The online version contains supplementary material available at 10.1186/s12859-022-04849-x.

## Background

Ewing sarcoma, characterized histologically by small, round sheets of blue-stained cells with prominent nuclei and sparse cytoplasm, is the second most common primary bone tumor mainly occurred in children, adolescents and young adult, which also can originate in soft tissues [1]. However, ES is a rare tumor with an incidence of about 1.5 cases per million in the world [2]. Meanwhile, it’s a lethal tumor with high rate of metastasis and high mortality. The relative 5-year survival rate reported by Timothy A. Damron et al. is 50.6%, lower than osteosarcoma and chondrosarcoma [1]. Notably, the 5-year overall survival rate of patients with metastases is lower than 30% and up to 20–25% of patients present with metastases at diagnosis [3]. Meanwhile, patients who relapse have a dismal prognosis, especially relapse within 2 years after diagnosis, the 5-year survival rate was even less than 10% [4]. Despite the progresses have been made in the treatment of ES, but the long-term survival rates of metastatic and relapsed patients have not improved significantly [2, 3]. Therefore, how to further improve the prognoses of the ES patients remains to be further studied and efforts on finding new molecular targets and new therapies for the existing targets are still ongoing.

Autophagy related genes (ARGs) in human referred to the human genes described so far as involved in autophagy and 222 ARGs were collected in the Human Autophagy Database to date (http://www.autophagy.lu/). As is known that autophagy is a self-degradation process of cytoplasmic proteins and damaged organelles precisely regulated by ARGs, which can prevent cell damage, promote cell survival in the absence of nutrients, respond to cytotoxic stimuli and etc. [5, 6]. It’s mainly activated by endoplasmic reticulum stress, low energy, nutrient starvation, hypoxia and reactive oxygen species [5, 6]. Obviously, autophagy acts as a self-protection mechanism of cells under physiological conditions [5, 6]. However, this survival mechanism also can help tumor cells to survive and disseminate under stress conditions [5–9]. Besides, many drug resistances of cancers are associated with autophagy as well [10, 11]. It’s reported that autophagy plays a dual role in human health and diseases [5, 6]. Generally, autophagy tended to function as a tumor suppressor mechanism in the early stage of tumorigenesis, while on the late stage of tumor, it can promote tumor proliferation, aggressiveness and metastasis [7–9]. Nevertheless, the controversial role of autophagy has also been reported in ES [12, 13]. Then, what role does autophagy play in the prognosis of ES deserves further study. Thus, in this study, we intend to explore this issue by bioinformatic tools and bridge the gap in this area. The findings of it may also indicate potential prognostic biomarkers and therapeutic targets in ES.

Therefore, in this study we intend to preliminarily explore the association between potential protective ARGs that with better prognosis in high expression group in KM analysis and the prognosis of ES, so as to find some potential prognostic biomarkers and even therapy targets for ES. Currently, several prognostic models have been reported in ES [14–17]. They explored the prognostic value of genes related to some other processes in ES, whereas no model has been established by ARGs, together with their roles in the prognosis of ES have not been explored, either. Therefore, it’s necessary to bridge the gap in this area. In addition, our study is also a supplement of the existing models, which can deepen our understanding of ES from a new perspective.

## Results

### Clinical characteristics of the enrolled ES patients

Data of 64 and 46 ES samples were extracted from GSE17679 and GSE65155 respectively. The former was set as training set and the latter was set as validation set. The clinical characteristics of the two sets showed that the characteristics of age and gender were similar between the two sets (Table 1).

**Table 1:** Clinicopathological characteristics in training and validation sets

Characteristics	GSE17679 (Training set)	GSE63155 (Validation set)
Age, mean ± SD	18.47 ± 6.69	12.29 ± 4.61
Gender, no. (%)		
Female	20 (31%)	19 (41%)
Male	44 (69%)	27 (59%)
Overall survival time, median (interquartile range)	38.65 (16.05–67.3)	60.32 (52.04–77.29)
Overall survival status, no. (%)		
Alive	24 (38%)	32 (70%)
Dead	40 (62%)	14 (30%)

### Potential protective ARGs screened by survival analysis

**Fig. 1:**
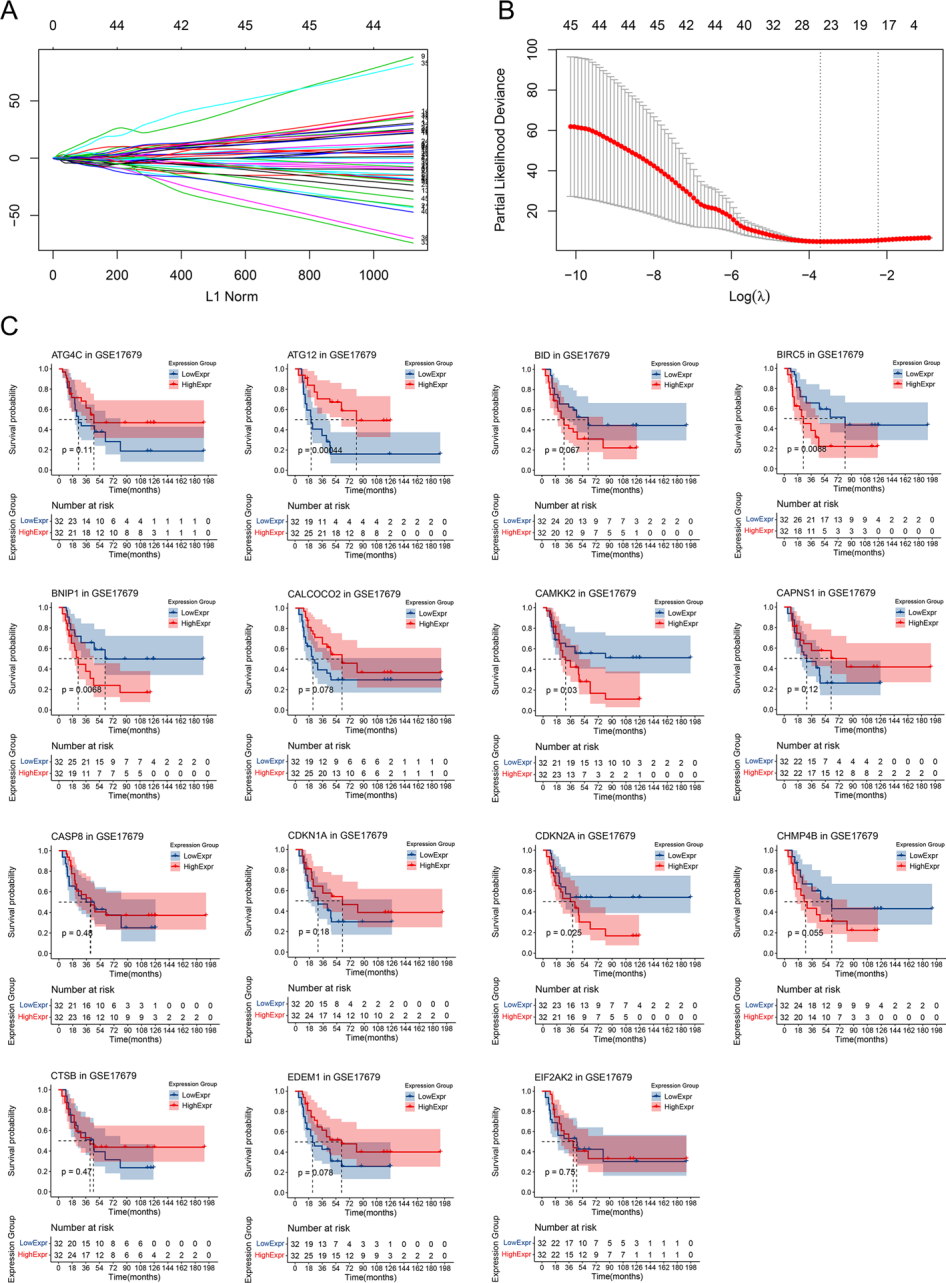
Screening protective ARGs with prognostic value by LASSO Cox regression analysis and KM analysis. **A** The LASSO coefficient profiles for the 46 genes in the tenfold cross-validations. **B** Partial likelihood deviance with changing of log (λ) plotted by LASSO regression in tenfold cross-validations. **C** KM plots for part of the ARGs screened by LASSO

**Fig. 2:**
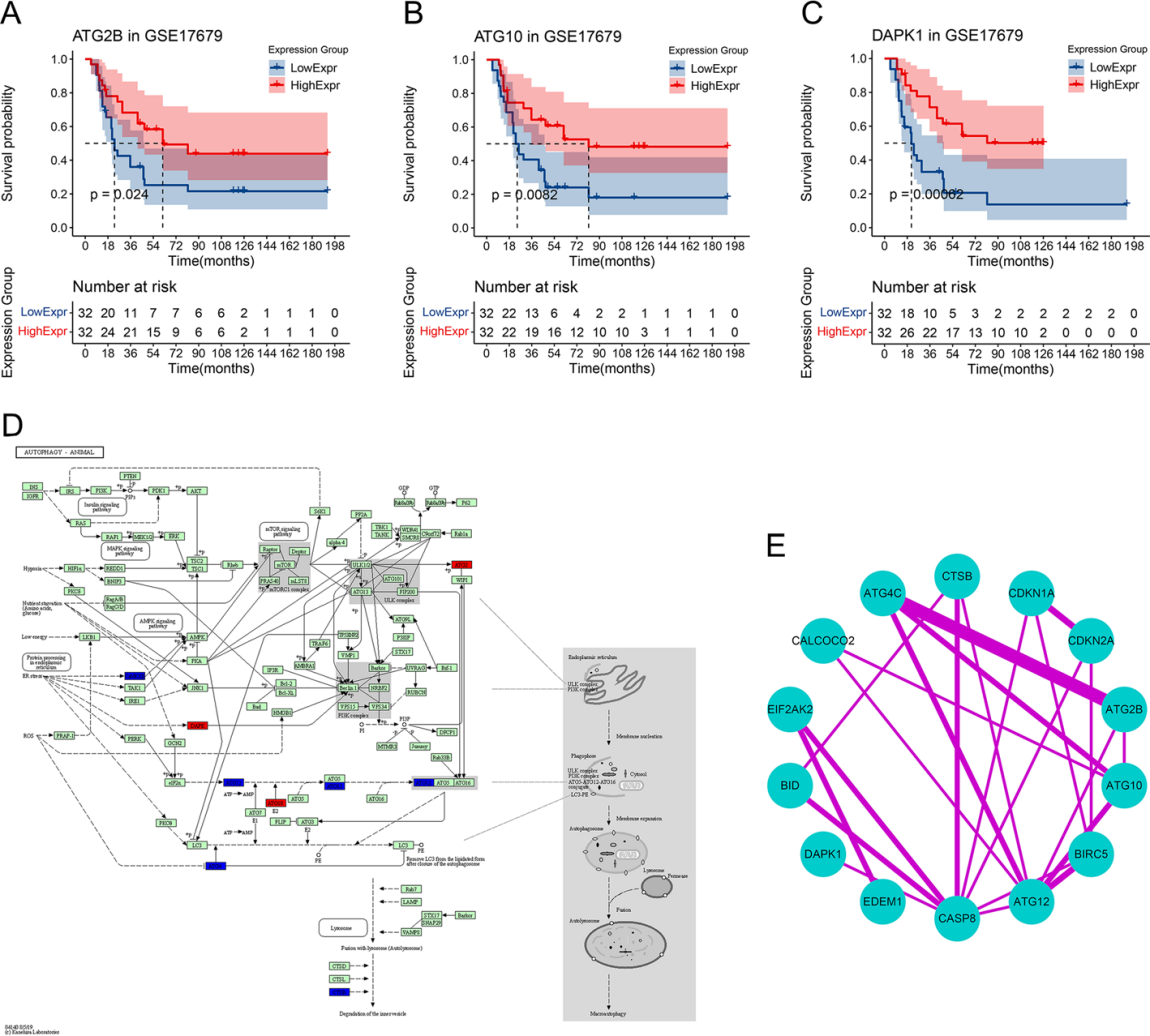
KM analysis, pathway map and PPI network analysis for gene screening. KM plots for ATG2B (**A**), ATG10 (**B**) and DAPK1 (**C**) in training set: x-axis referred to living time (months), y-axis referred to the survival probability (up) and  the group (down), number table in the lower part of the plot referred to number of patients at risk. (**D**) Autophagy pathway map (hsa04140) from KEGG website: red nodes referred to ATG2B, ATG10 and DAPK1, blue nodes were the other mapped ARGs screened by LASSO. (**E**) PPI network for proteins encoded by LASSO screened ARGs (interaction score ≥ 0.4): the width of the line referred to the co-expression strength between the two nodes

48 and 46 ARGs were screened by univariate (*P* value < 0.05) and multivariate Cox analyses (*P* value < 0.05), respectively (Additional file 1). 18 ARGs were retained by LASSO Cox regression analysis (λ = lambda.1se) (Fig. 1A, B). Then, KM analyses were performed for the 18 ARGs to select potential protective ARGs in training cohort (Figs. 1C, 2A–C). Results showed that *ATG2B*, *ATG10*, *ATG12* and *DAPK1* were ARGs with better prognosis in high expression group (*P* value < 0.05) (Fig. 2A–C). Finally, *ATG2B*, *ATG10* and *DAPK1* were selected as final AGRs most related to survival with *P* value < 0.01 in univariate Cox analyses. Map of autophagy pathway in human (hsa04140) were downloaded from KEGG website. 18 ARGs screened by LASSO were mapped to the autophagy pathway map, only 9 were successfully mapped (the red and blue nodes in the map, the red nodes referred to the final ARGs) (Fig. 2D). As was shown in Fig. 2D, *ATG2-WIPI* complex was involved in the processes from initiation to elongation of the autophagosome directly or by promoting *ATG12-ATG-ATG16* complex formation. *ATG10* acted as a E2 for *ATG12* in promoting *ATG12-ATG5-ATG16* complex formation. *DAPK* could promote the phosphorylation of the Beclin-1 playing an important role in autophagy. Lastly, PPI network showed the interactions among the proteins encoded by genes screened by LASSO and it could be seen that proteins encoded by *ATG2B*, *ATG10* and *DAPK1* were important nodes in this network, which in turn reflected their key roles in autophagy (Fig. 2E).

### Survival analysis for samples in different risk score groups

**Fig. 3:**
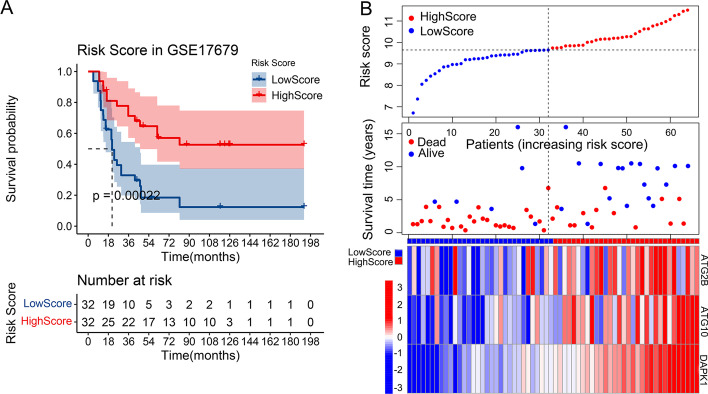
KM analysis for high and low risk score groups in training set and characteristics of samples in the two groups. **A** KM plot for high and low risk score groups in training set: x-axis referred to living time (months), y-axis referred to the survival probability (up) and risk score group (down), number table in the lower part of the plot referred to number of patients at risk. **B** Scatter plots for the survival characteristics of patients with increasing risk score and heatmap for the expression of final genes in patients with increasing risk score: the vertical dashed lines divided the samples into high and low score group, left side referred to high score group, right side referred to low score group

After risk scores were calculated and patients were divided into high and low risk score groups by median, KM analysis of the two group were performed. Result showed that there was significant difference between the low (blue curve) and high risk score (red curve) groups (*P* value = 0.00022) and patients in high score group got better outcomes in training set (Fig. 3A). The characteristics of samples from the two groups in training set were showed in Fig. 3B. The scatter plot in the middle of Fig. 3B showed that there were more blue dots in the upper left of the plot which means patients in high score group (left side of the plot) tended to have longer survival time, while more red dots in the bottom right of the plot which means patients in low score group (right side of the plot) tended to have shorter survival time. In sum, high risk score group tended to have better outcomes than low score group. Heatmap in Fig. 3B showed that *ATG2B*, *ATG10* and *DAPK1* tended to be highly expressed in high score group (Samples on the left side were high risk score group, right side were low risk score group. Blue color stood for low gene expression, red color stood for high gene expression in the heatmap).

### Evaluation of the prognostic model in training set

**Fig. 4:**
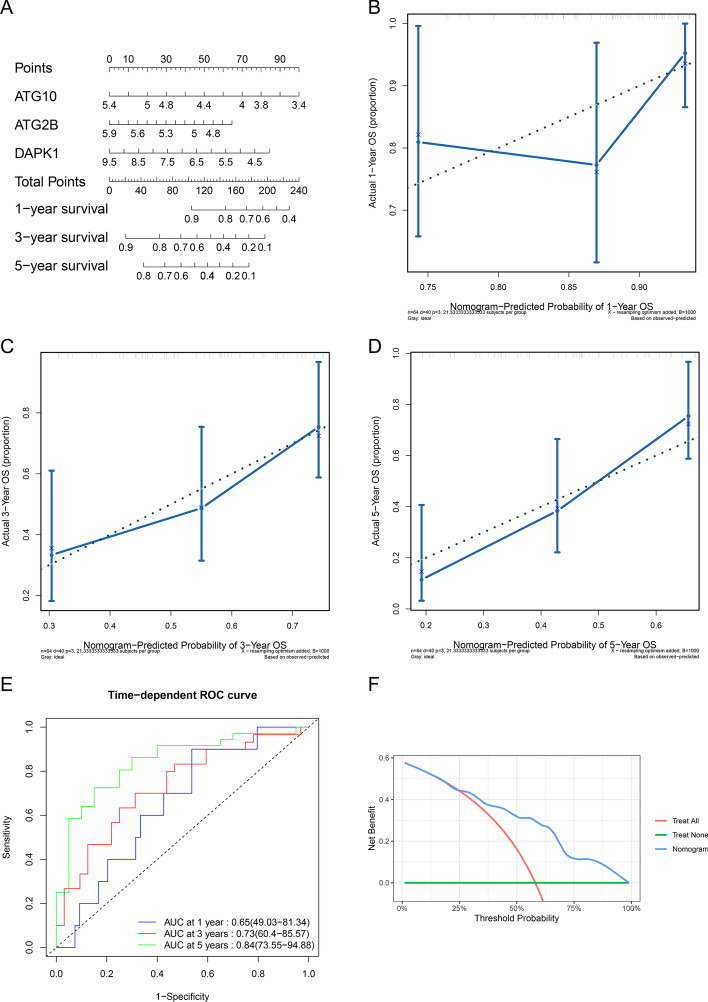
Establishing a prognostic model with final genes and evaluating it in training set. **A** Nomogram for the Cox proportional hazards model in GSE17679. Calibration of nomogram for 1-year (**B**), 3-year (**C**), 5-year (**D**) in GSE17679: x-axis referred to the predicted probability of overall survival by the model, y-axis referred to the actual probability of overall survival, the diagonal (dashed line) referred to the ideal status that the predicted survival rate equaled to the actual survival rate, the blue solid line referred to the actual status of the predicted and actual survival rate. (**E**) Time-dependent ROC curve of the model in GSE17679: x-axis equaled to 1—specificity of the model, y-axis was the sensitivity of the model. (**F**) 5-year DCA in GSE17679: x-axis referred to threshold probability for treatment or intervention, y-axis referred to the net benefit. Green line stood for no treatment for all samples, the net benefit was 0. Red line stood for treat all samples with the assumption of all samples would die within 5 years. Blue line stood for treat samples by the prediction of the model

A Cox proportional hazards model was established by *ATG2B*, *ATG10* and *DAPK1*, then visualized by a nomogram (Fig. 4A). An ES patient’s survival rate of 1-, 3- and 5-year could be predicted by just summing up the points of each final ARGs got and finding the corresponding rate in the scales at the bottom of the nomogram. The length of the lines for genes to some extent implied the importance of a gene in the model and obviously each of them played an important role in the model. C-index of the prognostic model in training set was 0.68 (95%CI: 0.63-0.72). Calibration analysis showed that the predicted 1-year overall survivals were close to the overall survivals observed and the predicted 3-,5-year overall survivals were in high agreement with the overall survivals observed, which indicated high accuracy of the model (Fig. 4B–D). Time-dependent ROC analysis showed that the 1-, 3-, 5-year AUCs were 0.65, 0.73, 0.84 respectively (Fig. 4E). The AUC of 1 year was a little bit low, but the 3- and 5- year AUCs of the model were excellent, which might imply a good long term predictive value for ES patients. Decision curve analysis (DCA) for 5-year prediction of the model indicated a higher net benefit than treat none and treat all plans (Fig. 4F).

### Validating the model in validation set

**Fig. 5:**
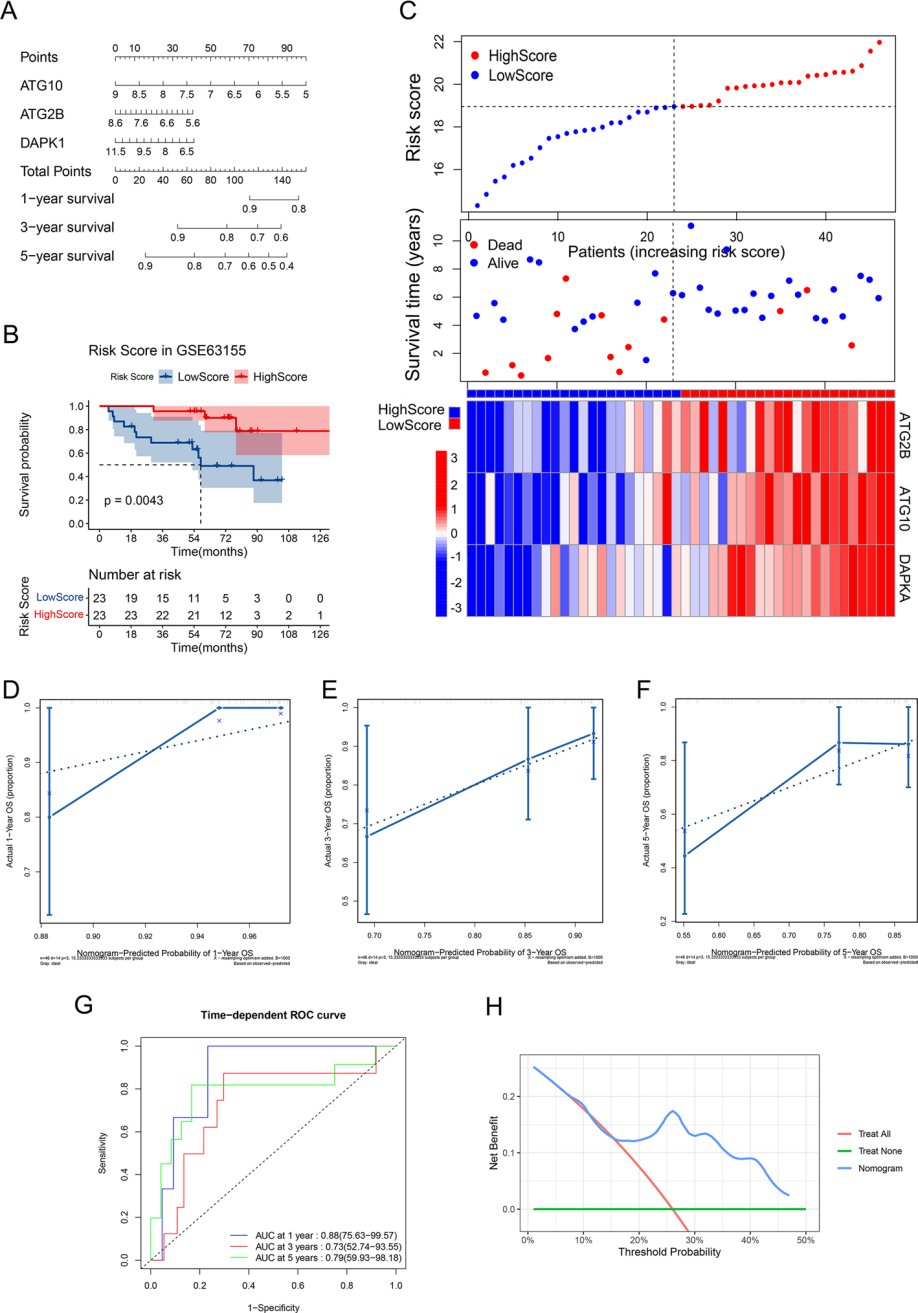
Validating the model in validation set. **A** Nomogram for the Cox proportional hazards model in GSE63155. **B** KM plot for high and low risk score groups in GSE63155. **C** Scatter plots for the survival characteristics of patients with increasing risk score and heatmap for the expression of final genes in patients with increasing risk score: the vertical dashed lines divided the samples into high and low score group, left side referred to high score group, right side referred to low score group. Calibration of nomogram for 1-year (**D**), 3-year (**E**), 5-year (**F**) in GSE63155: x-axis referred to the predicted probability of overall survival by the model, y-axis referred to the actual probability of overall survival, the diagonal (dashed line) referred to the ideal status that the predicted survival rate equaled to the survival rate, the blue solid line referred to the actual status of the predicted and actual survival rate. **G** Time-dependent ROC curve of the model in GSE63155: x-axis equaled to 1—specificity of the model, y-axis was the sensitivity of the model. (**H**) 5-year DCA in GSE63155: x-axis referred to threshold probability for treatment or intervention, y-axis referred to the net benefit. Green line stood for no treatment for all samples, the net benefit was 0. Red line stood for treating all samples with the assumption of all samples would die within 5 years. Blue line stood for treating samples by the prediction of the model

Nomogram for validation set (GSE63155) was shown in Fig. 5A. *ATG10*, with the longest line in nomogram, played the most important role in the model, which was also seen in training set. KM plot (Fig. 5B) indicated that patients in high risk score group (red curve) got a significant better outcomes than that in low score group (blue curve) (*P* value = 0.0043). Meanwhile, this was also seen in the middle plot of Fig. 5C: more alive patients with long survival time in high score group (more blue dots in the upper left of the plot). Heatmap of Fig. 5C showed that *ATG2B*, *ATG10* and *DAPK1* tended to be highly expressed in high score group (Samples on the left side were high risk score group, right side were low risk score group. Blue color stood for low gene expression, red color stood for high gene expression in the heatmap). C-index of the prognostic model in training set was 0.71 (95%CI: 0.63-0.78), even higher than that in training set. Calibration analysis showed the predicted 1-, 3-,5-year overall survivals by the model were in agreement with the overall survivals observed, which indicated its high prediction accuracy in validation set (Fig. 5D–F). Time-dependent ROC analysis showed that the 1-, 3-, 5-year AUCs were 0.88, 0.73, 0.79 respectively (Fig. 5G). The AUCs in the validation set were outstanding, they were even higher than that in training set, which suggested excellent prediction accuracy and discriminatory capacities of the model. Besides, DCA for 5-year prediction of the model also indicated a higher net benefit than the other two plans (Fig. 5H).

### Differential expression analysis of the final genes

**Fig. 6:**
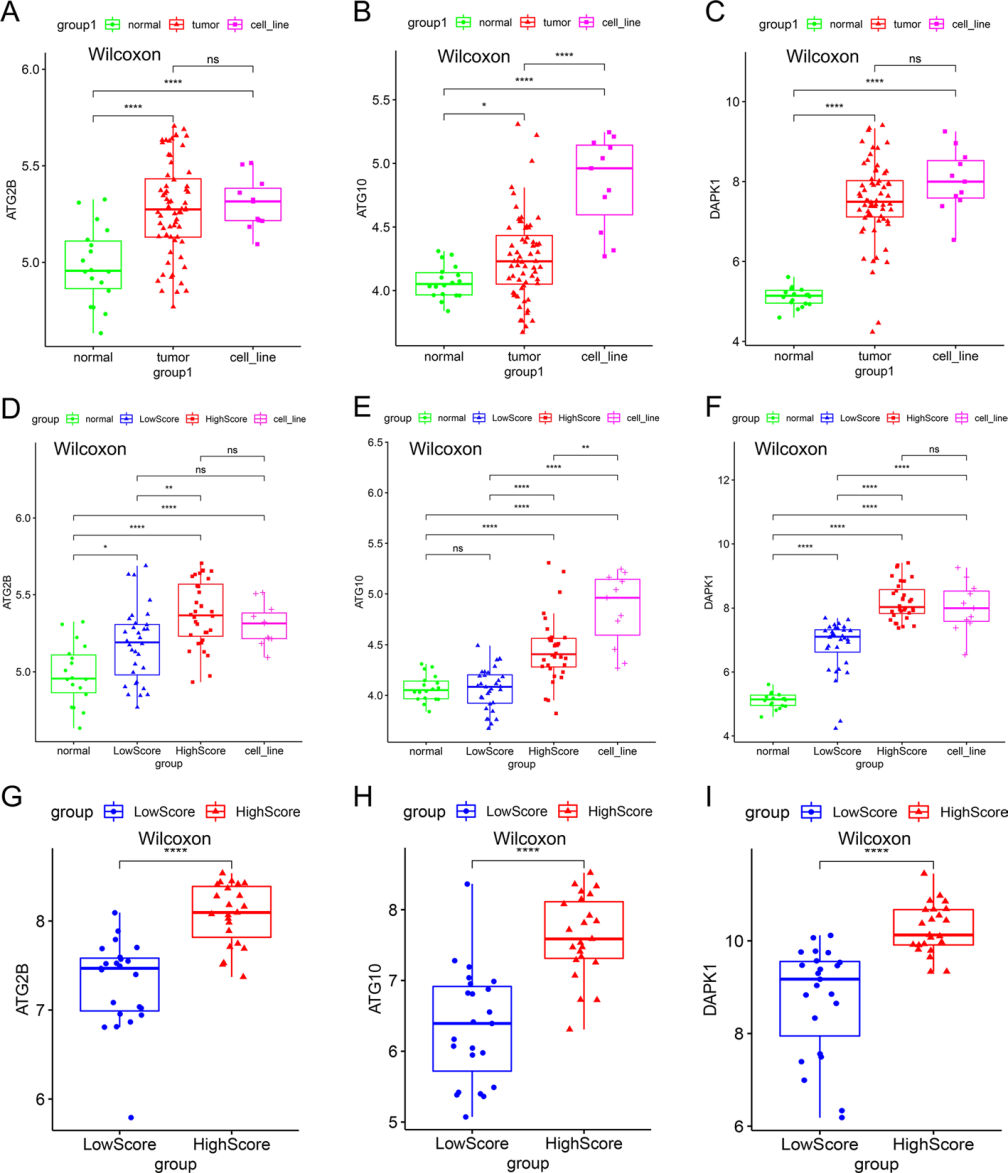
Expression of ATG2B, ATG10 and DAPK1 in different groups. Expression of ATG2B (**A**), ATG10 (**B**) and DAPK1 (**C**) in normal, ES cell line and tumor groups in training set; expression of ATG2B (**D**), ATG10 (**E**) and DAPK1 (**F**) in normal, ES cell line, low and high risk score groups in training set; expression of ATG2B (**G**), ATG10 (**H**) and DAPK1 (**I**) in low and high risk score groups in validation set: x-axis was group, y-axis was the expression of specific gene (p significance level: no significance (ns), p ≥ 0.05; *, p < 0.05; **, p < 0.01; ***, p < 0.001, ****, p < 0.0001)

Differential expressions of *ATG2B*, *ATG10* and *DAPK1* were explored between different groups in training and validation sets (Fig. 6A–I). Figure 6A showed that *ATG2B* was highly expressed in ES cell lines and tumor tissues and there was significant difference between normal and tumor samples (P < 0.01), but there were no significant differences between cell line and normal groups, cell line and tumor groups. The expressions of *ATG10* in tumor tissues was also higher than that in normal tissues (P < 0.05) (Fig. 6B), and it was extremely highly expressed in ES cell lines. Significant differences were found between cell line and normal, cell line and tumor groups (P < 0.0001). At the same time, differential expressions of *DAPK1* in these groups were similar to *ATG2B* (Fig. 6C). In Fig. 6D–F, ES tumor tissue group was divided into high and low risk score groups. Obviously, the expressions of the 3 genes in high score group were significantly higher than that in low score group (P < 0.0001). Meanwhile, we should notice that compared to high score group, the expressions of *ATG2B* and *ATG10* in low score group were closer to normal group, especially *ATG10*, the expressions of it were almost the same in the two groups (Fig. 6D, E). And compared to other groups, the expressions of the 3 genes in high score group were closer to the cell line group (Fig. 6D–F). Besides, compared to normal group, *DAPK1* was significantly highly expressed in both high and low score groups (Fig. 6F). In the validation set, the expressions of *ATG2B*, *ATG10* and *DAPK1* were found to be significantly highly expressed in high score groups (p < 0.0001) (Fig. 6G–I).

### Identification and functional analysis of the differentially expressed genes between high and low score groups

**Fig. 7:**
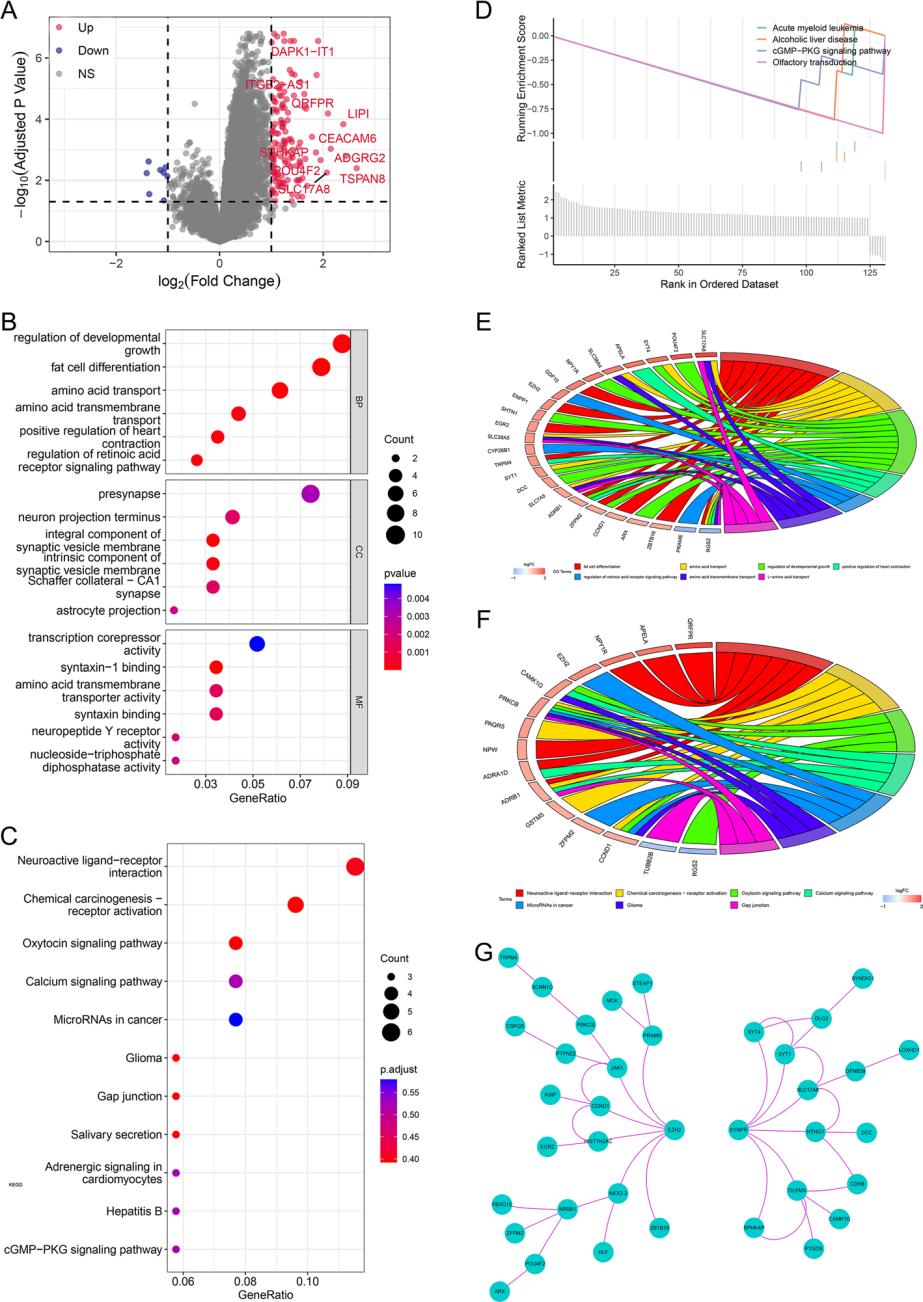
Differential analysis between high and low risk score groups and functional analyses of DEGs. **A** Volcano plot for DEGs between high and low risk score groups: x-axis referred to log_2_ Fold change of gene expression between the two groups, y-axis referred to − log_10_(adjusted *P* value) of gene expression between the two groups. red dots stood for significantly up regulated genes and blue dots stood for significantly down regulated genes in high risk score group, grey dots stood for non differentially expressed genes. **B** Dot plot for GO analysis of DEGs: x-axis referred to gene ratio, y-axis referred to clustered GO process. **C** Dot plot for KEGG analysis of DEGs: x-axis referred to gene ratio, y-axis referred to clustered KEGG process. **D** GSEA analysis for DEGs: x-axis referred ranked samples by enrichment score, y-axis referred to running enrichment score (up) and ranked list metric (down). **E** Chord plot for top 7 clustered GO terms: left side of the chord was the top7 clustered GO process, right side of the chord was the top DEGs by fold change. **F** Chord plot for top 7 clustered KEGG pathways: left side of the chord was the top7 clustered KEGG process, right side of the chord was the top DEGs by fold change. **G** PPI network of the DEGs

137 DEGs were screened between high and low score groups (Additional file 2), volcano plot was shown in Fig. 7A. GO clustering of the DEGs showed that the top 3 biological processes (BP) clustered were regulation of developmental growth, fat cell differentiation and amino acid transport, the top 3 cellular components (CC) clustered were presynapse, synaptic membrane, neuron projection terminus, the top 3 molecular functions (MF) clustered were transcription corepressor activity, syntaxin-1 binding, amino acid transmembrane transporter activity (Fig. 7B). The top 5 pathways clustered by KEGG were Neuroactive ligand-receptor interaction, Chemical carcinogenesis receptor activation, Oxytocin signaling pathway, Calcium signaling pathway, MicroRNAs in cancer (Fig. 7C). GSEA showed that the top 4 enriched gene sets: acute myeloid leukemia, alcoholic liver disease, cGMP-PKG signaling pathway, olfactory transduction were all down regulated in the high score group (Fig. 7D). GO chord plot showed that *BLC17A8, POU4F2, SYT4, APELA, SLC38A4, NPY1R, GDF10, EZH2, ENPP1, SHTN1, EGR2, SLC38A5, CYP26B1, TRPM4, SYT1, DCC, SLC7A5, ADRB1, ZFPM2, CCND1, ARX, ZBTB16, PRAME, RGS2* were involved in the top 7 GO terms (Fig. 7E). KEGG chord plot showed that *QRFPR, APELA, NPY1R, EZH2, CAMK1G, PRKCB, PAQR5, NPW, ADRA1D, ADRB1, GSTM5, ZFPM2, CCND1, TUBB2B and RGS2* were involved in the top 7 KEGG terms (Fig. 7F). PPI network showed that *EZH2* and *SYNPR* were hub genes involved in two independent processes respectively (Fig. 7G).

## Discussion

ES is a malignant tumor with high mortality, especially in patients with metastasis and recurrence. Recent development in treatment of ES has significantly improved the long-term survival in localized ES patients, from 10% in the era before chemotherapy to about 70% currently (5-year survival rate) [18–20]. However, the survival rates of patients with metastasis and recurrence are still unacceptable low [2]. To date, accumulating evidences suggest a close relationship between autophagy and ES [21–28]. Here, our finding that *ATG2B*, *ATG10* and *DAPK1* are potential protective ARGs with prognostic value in ES is valuable. It not only can deepen our insight into this area but may also contribute to the treatment of ES in future.

In our study, *ATG2B*, *ATG10* and *DAPK1* were strictly screened as the final potential protective ARGs by univariate and multivariate Cox regression, LASSO (Fig. 1A, B), KM analysis (Figs. 1C, 2A–C), pathway map analysis (Fig. 2D) and PPI network analysis (Fig. 2E) for the prognostic model of ES. The prediction accuracy and discriminatory capacities were accessed in both training and validation sets by KM analysis, risk score plots, C-index, calibration analysis, time-dependent ROC analysis and DCA. KM analysis showed that high risk score group got a better outcome than low score group (*P* < 0.05) (Figs. 3A, 5B). Distribution characteristics of high and low risk score group samples also confirmed that high score group tended to have longer survival time (Figs. 3B, 5C). Expression heatmap of the hubgenes indicated their high expression in high score group (Figs. 3B, 5C). The expression trend of the hubgenes were coincident with the risk score in samples. C-index was 0.68 in training set and 0.71 in validation set, both indicated high prediction accuracy of the model. The more expression of the hubgenes, the less points and the higher survival rate a patient got in nomograms (Figs. 4A, 5A) also supported the potential protective function of the hubgenes in ES. Calibration analysis (Figs. 4B–D, 5D–F) and time-dependent ROC analysis (Figs. 4E, 5G) all indicated high prediction accuracy of the model both in training and validation set. However, we noticed that the prediction accuracy of 1 year in training set was lower than that of 3- and 5- year (Figs. 4A, E, 5E). We considered this kind of fluctuation was mainly associated with two reasons here. Firstly, sequencing accuracy and sampling error including small sample size. We could see that the quality of the data in training set was inferior to that in the validation set by density plots. Secondly, differential expression of the genes in different stages and subtypes of tumor and their weight of role in the disease evolution. It’s true that some biomarkers worked well with long term survival, but with poor performance in short term survival prediction. The reliability of the model could be enhanced by improving the conditions above. In the end, DCA in training and validation sets indicated higher net benefits by the model than treat none and treat all strategy (Figs. 4F, 5H). In summary, the model established by the 3 potential protective hubgenes had high prediction accuracy and good applicability.

Moreover, the expression profiles of the 3 ARGs were explored in different groups in training and validation sets. First of all, we compared their expressions in normal tissues, ES cell lines and tumor tissues. Comparing to normal group, *ATG2B, ATG10, DAPK1* were all high expressed in tumor and cell lines (Fig. 6A–C). Meanwhile, the expressions in cell lines even higher than that in tumor tissues, which might be mainly due to the higher purity of tumor cells in cell lines. Secondly, comparison of their expressions between high and low risk score groups showed that they were all highly expressed in high score group both in training and validation sets (Fig. 6D-I). The interesting thing was that when compared to normal and cell line groups, the expressions in low score group tended to be closer to that in normal group, which suggested that in vivo ES cells might gain more aggression by inhibiting autophagy. A potential explanation for this might be that autophagy could lead to programmed cell death in tumor cells while low autophagy might add to the accumulation of malignant mutations [5–8].

In addition, in order to further explore the potential reasons for the difference in prognosis of the two score groups, differential and functional analyses of the two groups were performed. GO clustering of DEGs suggested that DEGs were mainly involved in membrane and transmembrane transport, which could be activated in autophagy process (Fig. 7B, E). Meanwhile, we noticed that Calcium signaling pathway and MicroRNAs in cancer were top 5 clustered pathways in KEGG (Fig. 7C, F). It had been reported that calcium signaling pathway was mainly involved in the regulations of apoptosis, autophagy and cell proliferation in cancers and any impairment to this function might result in low sensitivity to cell death inducers, thereby promoting tumor growth and metastasis [29]. While MicroRNAs were frequently considered as post-transcriptional regulators of gene expression and many key ARGs were also regulated by MicroRNAs [30, 31]. In addition, we also noticed that cGMP-PKG signaling pathway was enriched by GSEA (Fig. 7D). cGMP-PKG signaling pathway could inhibit apoptosis by decreasing the activity of caspase 3. While in our study, cGMP-PKG signaling pathway was down regulated in high score group which meant that the inhibition of apoptosis was released in that group and this might be also one of the reasons for the better prognosis in high score group. PPI network analysis of DEGs got two hub proteins, which might also contribute to the prognostic difference in the two groups (Fig. 7G). Firstly, *SYNPR,* an intrinsic membrane protein of small synaptic vesicles, *SYNPR* may be involved in autophagy through membrane transport. It’s also involved in the top GO and KEGG processes (Fig. 7E, F). Secondly, *EZH2* was upstream protein involved in cancer initiation, progression, metastasis, metabolism, drug resistance and immunity regulation [32]. Over expression of *EZH2* might associated with the metastasis and poor prognosis in ES, but the mechanism was still unclear and it’s not involved in the top GO and KEGG processes in our study [33–35].

Above all, our results suggested *ATG2B*, *ATG10* and *DAPK1* were potential protective ARGs with prognostic value in ES. They might affect the prognosis of ES by autophagy and interactions with other genes and processes directly or indirectly.

Firstly, *ATG2B*, autophagy related 2B, encoded a protein required for autophagy, which was involved in autophagosome formation. Mutation of this gene was reported to be associated with predisposition to myeloid malignancies (NCBI Reference Sequences (RefSeq)) [36, 37]. Besides, there were also many studies indicating that *ATG2B* was involved in many other tumors. Mi Ran Kang et al. proved that *ATG2B* mutation might contribute to cancer development by deregulating the autophagy process in gastric and colorectal carcinomas with high microsatellite instability [38]. Jiali Wei et al. revealed that cell proliferation was inhibited in non-small cell lung cancer cells by targeting *ATG2B* to inhibit autophagy [39, 40]. Xuemei Zhang et al. found that methylation of *ATG2B* CpG island promoter might be associated with the initiation and progression of breast carcinoma [41]. Xiaoqing Bi et al. reported that upregulation of *ATG2B* could inhibit cell proliferation and lead to cell apoptosis in cutaneous squamous cell carcinoma cells [42]. There were also many reports indicating that *ATG2B* was associated with drug resistance by activating autophagy in multiple tumors [10, 11]. Although *ATG2B* had not been reported in ES, but there had been plenty of reports that autophagy was associated with the prognosisi of ES [19–26]. In our study, *ATG2B* was found to be a potential protective factor in ES, the same as it's in gastric and colorectal carcinomas, breast carcinoma and cutaneous squamous cell carcinoma, but opposite to non-small cell lung cancer.

Secondly, *ATG10*, autophagy related 10, was an E2-like enzyme involved in the ubiquitin-like modification of ATG12-ATG5-ATG16 complex, which was essential for the elongation and maturation of autophagosomes. Meanwhile, *ATG10* was also found to play important roles in several tumors. It’s reported that potentially functional polymorphisms in *ATG10* were found to be associated with risk of breast cancer and acute myeloid leukemia [43, 44]. Hao Shen et al. declared that *ATG10* could inhibit tumor migration and invasion by activating autophagy and apoptosis in papillary thyroid carcinomas [45]. Qing-Hua Cao et al. analyzed 352 patients by bioinformatics methods finding that *ATG10* was a favorable prognostic factor for the overall survival in gastric cancer [46]. Yoon Kyung Jo et al. found that knock down of *ATG10* promoted cell migration and invasion of colorectal cancer cells [47]. Kaipeng Xie et al. revealed that high expression of *ATG10* leaded to short survival by facilitated tumor cell proliferation and migration in lung cancer. Herein, we found *ATG10* was a favorable factor in the prognosis of ES. The same effect was also found in papillary thyroid carcinomas, gastric cancer, colorectal cancer, while the opposite effect was seen in lung cancer.

Lastly, *DAPK1*, death associated protein kinase 1, was a positive mediator of gamma-interferon induced programmed cell death and a candidate for antioncogene (NCBI RefSeq). It’s act as a tumor suppressor in multiple cancers, such as lymphomas [48], invasive ductal carcinoma [49], gastrointestinal cancer [50], gastric cancer [51], liver cancer [52], etc. It was reported to suppress tumor genesis and progress by promoting autophagy and apoptosis and this was in coincidence with the conclusion reached by our study [53, 54].

Although the function of autophagy was complex and often contradictory in cancers, but there were many evidences supporting that upregulation of *ATG2B*, *ATG10*, *DAPK1* might contribute to good prognosis by promoting autophagy and apoptosis in ES. Conglin Ye et al. reported that the proliferation, invasion and migration of Ewing sarcoma cells were decreased by knocking down Beclin-1 which was required for the formation of autophagosomes [23]. Séverine Lorin et al. found that 2-methoxyestradiol could treat ES patients by enhancing autophagy and apoptosis through the activation of both p53 and JNK pathways [26, 27]. Mojgan Djavaheri-Mergny et al. demonstrated that autophagy might amplify apoptosis and stimulation of autophagy might be a potential way to bypass NF-kappaB induced drug resistance in ES [28].

Generally, *ATG2B*, *ATG10*, *DAPK1* were found to be potential protective ARGs with prognostic value in ES and established a prognostic model. The model was successfully validated in an independent external cohort with excellent prediction accuracy and discriminatory capacities. The potential cause of the different prognoses between high and low score groups was that they might affect the apoptosis and malignance of ES cells through autophagy and crosstalk with other processes.

In the end, some limitations of this study should be noticed. For one thing, because ES is a rare tumor, data with large sample size are not available. For another thing, our conclusion is only validated in the existing public datasets and literature.

## Conclusions

*ATG2B*, *ATG10* and *DAPK1* were autophagy related genes with potential protective function in ES. The prognostic model established by them exhibited excellent prediction accuracy and discriminatory capacities. They might be used as potential prognostic biomarkers and therapeutic targets in ES.

## Methods

### Data collection

The Gene Expression Omnibus (GEO) (https://www.ncbi.nlm.nih.gov/geo/) is an international public repository that provides microarray, next-generation sequencing and other forms of high-throughput functional genomics data submitted by the research community. In this study, we searched GEO website for datasets with RNA expression data of ES tissue specimens, corresponding survival data of patients and including at least 40 ES samples (March 27 2022). Then, only GSE17679 and GSE63155 were qualified. GSE17679 included normalized RNA array data by Affymetrix Human Genome U133 Plus 2.0 Array and survival data of 64 samples uploaded by university of Helsinki, Finland, on Aug 17, 2009 [55]. GSE63155 include normalized RNA array data by Affymetrix Human Exon 1.0 ST Array [transcript (gene) version] and survival data of 46 samples uploaded by university of Michigan, USA, on Nov 10, 2014 [56]. Meanwhile, GSE17679 also contained RNA expression data of 11 in vitro cultured Ewing sarcoma cell lines (GSM439930-GSM439940: Ewing sarcoma cell line 6647, IOR-BRZ, IOR-BRZ, IOR-CLB, IOR-NGR, IOR-RCH, LAP35, RDES, SKES1, SKNMC, TC71) and 18 normal muscle tissue RNA expression data measured by the same method [55]. RNA expression data from GSE17679 and GSE63155 had already been normalized by RMA method and quantile method, respectively. Quality control was performed by box plots (Additional file 3A-B) and density plots (Additional file 3C-D). Results showed that the expression data were well normalized and comparable in each set with high quality and no outlier. 222 ARGs were downloaded from the Human Autophagy Database (http://www.autophagy.lu/) (March 27 2022).

### Identify ARGs with prognostic significance by univariate and multivariate Cox analyses and LASSO Cox regression analysis

Only common ARGs expressed both in GSE17679 and GSE63155 datasets were selected for survival analyses in case ARGs identified in training set could not be validated in validation set. In our study, GSE17679 cohort was set as training set, the other validation set. “survival” package was adopted to integrate survival data of samples for Cox analysis [57]. Then, univariate Cox regression analysis were used to screen genes with prognostic value. Genes with *P* value < 0.05 in univariate Cox regression analysis were further screened by multivariate Cox regression analysis (*P* value < 0.05). Thereafter, genes filtered by multivariate Cox regression analysis were further screened again by the least absolute shrinkage and selection operator (LASSO) Cox regression analysis with 10 times cross validation (λ = lambda.1se) [58].

### Screen potential protective ARGs by KM analysis and explore their roles in autophagy

Then, KM analysis was used to explore the prognostic value of each retained gene by dividing patients into high and low expression groups with median expression of the gene. Genes with better prognosis in high expression group in KM analyses (*P* value < 0.05) and meanwhile with *P* value < 0.01 in univariate Cox regression analysis were selected as final potential protective ARGs for a prognostic model in ES. Autophagy pathway map was downloaded from KEGG website (https://www.genome.jp/kegg/) and protein protein interact (PPI) network analysis for proteins encoded by genes screened by LASSO were also employed to explore their roles in autophagy (interaction score ≥ 0.4).

### Risk score calculation and survival analysis for samples in different groups

Risk scores for samples were calculated by sum up the risk score of each final ARG and risk score for a gene was calculated by multiplying its expression value by its coefficient value in multivariate Cox regression model (Formula: $R{\text{isk}}\,score = \sum\nolimits_{{{\text{i}} = {1}}}^{{\text{n}}} {Exp_{{gene_{i} }} \times } \, coefficient_{{gene_{i} }}$, Exp _gene i_ referred to the expression of gene i, coefficient _gene i_ referred to the coefficient value of gene i, n referred to the number of genes involved in the model). Then, samples were divided into low and high risk score group by median of the risk scores for samples. Thereafter, KM analysis was used to investigate the prognosis of the two groups. Meanwhile, scatter plots and heatmap were used to exhibit the distribution characteristics of samples and hubgenes in the two groups.

### Establish a prognostic model and evaluate it in training set

Final potential protective ARGs were used to build a prognostic model. Nomogram was used to visualize the Cox proportional hazards model. Then, the prediction accuracy and discriminatory capacities were assessed by C-index, calibration analysis, time-dependent ROC analysis and DCA in GSE17679.

### Validate the model in validation set

The prediction accuracy and discriminatory capacities of the model were also validated in GSE63155 by KM analysis, risk score plots, C-index, calibration analysis, time-dependent ROC analysis and DCA.

### Differential expression analysis of the final ARGs

Differential expression analysis of the final genes was investigated in GSE17679 between normal tissue, ES cell line, low and high risk score groups by Wilcoxon method after removing batch effect by ComBat function of “sva” package and *P* value < 0.05 was considered statistically significant. Meanwhile, differential expression analysis was also performed in GSE63155 between low and high risk score groups but without removing batch effect (no batch effect in GSE63155).

### Identification and functional analysis of the differentially expressed genes between high and low score groups

“limma” package was used to identify the differentially expressed genes (DEGs) between the high and low risk score groups, Benjamini and Hochberg method was used to adjust the *P* value (adjusted *P* value < 0.05, |log_2_FC| > 1) [59]. Then, GO and KEGG clustering, gene set enrichment analysis (GSEA), PPI network analysis were preformed to explore their functional enrichment and interactions (interaction score ≥ 0.4).

### Statistical analysis

In this study, R software v3.63 was used to process data and generate charts. PPI network analyses were explored on STRING website (interaction score ≥ 0.4) (https://cn.string-db.org/) and visualized by Cytoscape software v3.7.1. Flexible statistical methods were adopted for the statistical analysis. The work flow of this study was shown in Additional file 4.

## Supplementary Information

**Additional file 1**. Univariate and multivariate Cox analyses for ARGs.

**Additional file 2**. DEGs identified by “limma” between high and low score groups.

**Additional file 3.** Box plots and density plots for gene expression profile of samples. Box plots for gene expression in GSE17679 (**A**) and GSE63155 (**B**): x-axis was the samples, y-axis was the expression of gene. Density plots for gene expression in GSE17679 (**C**) and GSE63155 (**D**): x-axis referred to the gene intensity, y-axis referred to the expression density of genes converted by log2.

**Additional file 4.** Flow chart of this study.

## Data Availability

As stated in methods, all the original data were downloaded from public databases. Expression and survival data of GSE17679 and GSE 63155 were downloaded from the Gene Expression Omnibus (GEO) dataset (https://www.ncbi.nlm.nih.gov/geo/). 222 autophagy-related genes (ARGs) were downloaded from the Human Autophagy Database (March 27 2022) (http://www.autophagy.lu/).

## Abbreviations

ARG: Autophagy related gene
BP: Biological process
CC: Cellular component
CI: Confidence interval
DCA: Decision curve analysis
DEGs: Differentially expressed genes
ES: Ewing sarcoma
GEO: Gene expression omnibus database
GO: Gene ontology
GSEA: Gene set enrichment analysis
KEGG: Kyoto Encyclopedia of Genes and Genomes
KM: Kaplan-Meier
LASSO: The least absolute shrinkage and selection operator
MF: Molecular function
OS: Overall survival
PPI: Protein protein interact
RefSeq: Reference Sequences

## References

[1] Damron TA, Ward WG, Stewart A (2007). Osteosarcoma, chondrosarcoma, and Ewing's sarcoma: National Cancer Data Base Report. Clin Orthop Relat Res.

[2] Grunewald TGP, Cidre-Aranaz F, Surdez D, Tomazou EM, de Alava E, Kovar H (2018). Ewing sarcoma. Nat Rev Dis Primers.

[3] Gaspar N, Hawkins DS, Dirksen U, Lewis IJ, Ferrari S, Le Deley MC (2015). Ewing sarcoma: current management and future approaches through collaboration. J Clin Oncol.

[4] Stahl M, Ranft A, Paulussen M, Bölling T, Vieth V, Bielack S (2011). Risk of recurrence and survival after relapse in patients with Ewing sarcoma. Pediatr Blood Cancer.

[5] Choi AM, Ryter SW, Levine B (2013). Autophagy in human health and disease. N Engl J Med.

[6] Mizushima N, Levine B (2020). Autophagy in human diseases. N Engl J Med.

[7] Glick D, Barth S, Macleod KF (2010). Autophagy: cellular and molecular mechanisms. J Pathol.

[8] Li X, He S, Ma B (2020). Autophagy and autophagy-related proteins in cancer. Mol Cancer.

[9] Mowers EE, Sharifi MN, Macleod KF (2018). Functions of autophagy in the tumor microenvironment and cancer metastasis. Febs J.

[10] Nawrocki ST, Wang W, Carew JS (2020). Autophagy: new insights into its roles in cancer progression and drug resistance. Cancers (Basel).

[11] Smith AG, Macleod KF (2019). Autophagy, cancer stem cells and drug resistance. J Pathol.

[12] Koustas E, Sarantis P, Karamouzis MV, Vielh P, Theocharis S (2021). The controversial role of autophagy in Ewing sarcoma pathogenesis-current treatment options. Biomolecules.

[13] Levy JMM, Towers CG, Thorburn A (2017). Targeting autophagy in cancer. Nat Rev Cancer.

[14] Chen Y, Su H, Su Y, Zhang Y, Lin Y, Haglund F (2021). Identification of an RNA-binding-protein-based prognostic model for Ewing sarcoma. Cancers (Basel).

[15] Chen ZY, Yang H, Bu J, Chen Q, Yang Z, Li H (2021). Prognosis implication of a novel metabolism-related gene signature in Ewing sarcoma. J Oncol.

[16] Fu Z, Yu B, Liu M, Wu B, Hou Y, Wang H (2021). Construction of a prognostic signature in Ewing's sarcoma: based on metabolism-related genes. Transl Oncol.

[17] Jiang J, Zhan X, Xu G, Liang T, Yu C, Liao S (2021). Glycolysis- and immune-related novel prognostic biomarkers of Ewing's sarcoma: glucuronic acid epimerase and triosephosphate isomerase 1. Aging (Albany NY).

[18] Ludwig JA, Meyers PA, Dirksen U (2021). Ewing's sarcoma. N Engl J Med.

[19] Balamuth NJ, Womer RB (2010). Ewing's sarcoma. Lancet Oncol.

[20] Blay JY, De Pinieux G, Gouin F (2021). Ewing's sarcoma. N Engl J Med.

[21] Lu Q, Zhang Y, Ma L, Li D, Li M, Li J (2017). EWS-FLI1 positively regulates autophagy by increasing ATG4B expression in Ewing sarcoma cells. Int J Mol Med.

[22] Lu Q, Zhang Y, Ma L, Li D, Li M, Liu P (2019). TRIM3 negatively regulates autophagy through promoting degradation of Beclin1 in Ewing sarcoma cells. Onco Targets Ther.

[23] Ye C, Yu X, Liu X, Zhan P, Nie T, Guo R (2018). Beclin-1 knockdown decreases proliferation, invasion and migration of Ewing sarcoma SK-ES-1 cells via inhibition of MMP-9. Oncol Lett.

[24] Kim Y, Kang YS, Lee NY, Kim KY, Hwang YJ, Kim HW (2015). Uvrag targeting by Mir125a and Mir351 modulates autophagy associated with Ewsr1 deficiency. Autophagy.

[25] Patel M, Gomez NC, McFadden AW, Moats-Staats BM, Wu S, Rojas A (2014). PTEN deficiency mediates a reciprocal response to IGFI and mTOR inhibition. Mol Cancer Res.

[26] Lorin S, Pierron G, Ryan KM, Codogno P, Djavaheri-Mergny M (2010). Evidence for the interplay between JNK and p53-DRAM signalling pathways in the regulation of autophagy. Autophagy.

[27] Lorin S, Borges A, Ribeiro Dos Santos L, Souquère S, Pierron G, Ryan KM (2009). c-Jun NH2-terminal kinase activation is essential for DRAM-dependent induction of autophagy and apoptosis in 2-methoxyestradiol-treated Ewing sarcoma cells. Cancer Res.

[28] Djavaheri-Mergny M, Amelotti M, Mathieu J, Besançon F, Bauvy C, Souquère S (2006). NF-kappaB activation represses tumor necrosis factor-alpha-induced autophagy. J Biol Chem.

[29] Patergnani S, Danese A, Bouhamida E, Aguiari G, Previati M, Pinton P (2020). Various aspects of calcium signaling in the regulation of apoptosis, autophagy, cell proliferation, and cancer. Int J Mol Sci.

[30] Krol J, Loedige I, Filipowicz W (2010). The widespread regulation of microRNA biogenesis, function and decay. Nat Rev Genet.

[31] Kim Y, Lee J, Ryu H (2015). Modulation of autophagy by miRNAs. BMB Rep.

[32] Duan R, Du W, Guo W (2020). EZH2: a novel target for cancer treatment. J Hematol Oncol.

[33] Kailayangiri S, Altvater B, Lesch S, Balbach S, Göttlich C, Kühnemundt J (2019). EZH2 inhibition in Ewing sarcoma upregulates G(D2) expression for targeting with gene-modified T cells. Mol Ther.

[34] Ramaglia M, D'Angelo V, Iannotta A, Di Pinto D, Pota E, Affinita MC (2016). High EZH2 expression is correlated to metastatic disease in pediatric soft tissue sarcomas. Cancer Cell Int.

[35] Richter GH, Plehm S, Fasan A, Rössler S, Unland R, Bennani-Baiti IM (2009). EZH2 is a mediator of EWS/FLI1 driven tumor growth and metastasis blocking endothelial and neuro-ectodermal differentiation. Proc Natl Acad Sci USA.

[36] Pegliasco J, Hirsch P, Marzac C, Isnard F, Meniane JC, Deswarte C (2022). Germline ATG2B/GSKIP-containing 14q32 duplication predisposes to early clonal hematopoiesis leading to myeloid neoplasms. Leukemia.

[37] Saliba J, Saint-Martin C, Di Stefano A, Lenglet G, Marty C, Keren B (2015). Germline duplication of ATG2B and GSKIP predisposes to familial myeloid malignancies. Nat Genet.

[38] Kang MR, Kim MS, Oh JE, Kim YR, Song SY, Kim SS (2009). Frameshift mutations of autophagy-related genes ATG2B, ATG5, ATG9B and ATG12 in gastric and colorectal cancers with microsatellite instability. J Pathol.

[39] Wei J, Ma Z, Li Y, Zhao B, Wang D, Jin Y (2015). miR-143 inhibits cell proliferation by targeting autophagy-related 2B in non-small cell lung cancer H1299 cells. Mol Med Rep.

[40] Li Y, Zhang H, Guo J, Li W, Wang X, Zhang C (2021). Downregulation of LINC01296 suppresses non-small-cell lung cancer via targeting miR-143-3p/ATG2B. Acta Biochim Biophys Sin (Shanghai).

[41] Zhang X, Li C, Wang D, Chen Q, Li CL, Li HJ (2016). Aberrant methylation of ATG2B, ATG4D, ATG9A and ATG9B CpG island promoter is associated with decreased mRNA expression in sporadic breast carcinoma. Gene.

[42] Bi X, Jiang Z, Luan Z, Qiu D (2021). Crocin exerts anti-proliferative and apoptotic effects on cutaneous squamous cell carcinoma via miR-320a/ATG2B. Bioengineered.

[43] Castro I, Sampaio-Marques B, Areias AC, Sousa H, Fernandes Â, Sanchez-Maldonado JM (2021). Functional genetic variants in ATG10 are associated with acute myeloid leukemia. Cancers (Basel).

[44] Qin Z, Xue J, He Y, Ma H, Jin G, Chen J (2013). Potentially functional polymorphisms in ATG10 are associated with risk of breast cancer in a Chinese population. Gene.

[45] Shen H, Lin Z, Shi H, Wu L, Ma B, Li H (2020). MiR-221/222 promote migration and invasion, and inhibit autophagy and apoptosis by modulating ATG10 in aggressive papillary thyroid carcinoma. 3 Biotech.

[46] Cao QH, Liu F, Yang ZL, Fu XH, Yang ZH, Liu Q (2016). Prognostic value of autophagy related proteins ULK1, Beclin 1, ATG3, ATG5, ATG7, ATG9, ATG10, ATG12, LC3B and p62/SQSTM1 in gastric cancer. Am J Transl Res.

[47] Jo YK, Roh SA, Lee H, Park NY, Choi ES, Oh JH (2017). Polypyrimidine tract-binding protein 1-mediated down-regulation of ATG10 facilitates metastasis of colorectal cancer cells. Cancer Lett.

[48] Özdemir İ, Pınarlı FG, Pınarlı FA, Aksakal FNB, Okur A, Uyar Göçün P (2018). Epigenetic silencing of the tumor suppressor genes SPI1, PRDX2, KLF4, DLEC1, and DAPK1 in childhood and adolescent lymphomas. Pediatr Hematol Oncol.

[49] Ghalkhani E, Akbari MT, Izadi P, Mahmoodzadeh H, Kamali F (2021). Assessment of DAPK1 and CAVIN3 gene promoter methylation in breast invasive ductal carcinoma and metastasis. Cell J.

[50] Yuan W, Chen J, Shu Y, Liu S, Wu L, Ji J (2017). Correlation of DAPK1 methylation and the risk of gastrointestinal cancer: a systematic review and meta-analysis. PLoS ONE.

[51] Guo Z, Zhou C, Zhou L, Wang Z, Zhu X, Mu X (2022). Overexpression of DAPK1-mediated inhibition of IKKβ/CSN5/PD-L1 axis enhances natural killer cell killing ability and inhibits tumor immune evasion in gastric cancer. Cell Immunol.

[52] Li L, Guo L, Wang Q, Liu X, Zeng Y, Wen Q (2017). DAPK1 as an independent prognostic marker in liver cancer. PeerJ.

[53] Movahhed P, Saberiyan M, Safi A, Arshadi Z, Kazerouni F, Teimori H (2022). The impact of DAPK1 and mTORC1 signaling association on autophagy in cancer. Mol Biol Rep.

[54] Singh P, Ravanan P, Talwar P (2016). Death Associated Protein Kinase 1 (DAPK1): a regulator of apoptosis and autophagy. Front Mol Neurosci.

[55] Savola S, Klami A, Myllykangas S, Manara C, Scotlandi K, Picci P (2011). High expression of complement component 5 (C5) at tumor site associates with superior survival in Ewing's sarcoma family of tumour patients. ISRN Oncol.

[56] Volchenboum SL, Andrade J, Huang L, Barkauskas DA, Krailo M, Womer RB (2015). Gene expression profiling of Ewing sarcoma tumors reveals the prognostic importance of tumor-stromal interactions: a report from the children's oncology group. J Pathol Clin Res.

[57] Groeneveld CS, Chagas VS, Jones SJM, Robertson AG, Ponder BAJ, Meyer KB (2019). RTNsurvival: an R/Bioconductor package for regulatory network survival analysis. Bioinformatics.

[58] Tibshirani R (1997). The lasso method for variable selection in the Cox model. Stat Med.

[59] Ritchie ME, Phipson B, Wu D, Hu Y, Law CW, Shi W (2015). limma powers differential expression analyses for RNA-sequencing and microarray studies. Nucleic Acids Res.

