# Supplementary material for: The prognostic value of autophagy related genes with potential protective function in Ewing sarcoma
Source: BMC Bioinformatics. 2022 Jul 28;23:306. doi: 10.1186/s12859-022-04849-x (PMC9335970; doi:10.1186/s12859-022-04849-x)
Supplement: Supplementary file 1 — Additional file 1. Univariate and multivariate Cox analyses for ARGs. [file 12859_2022_4849_MOESM1_ESM.docx]

**Additional file 1** Univariate and multivariate Cox analyses for ARGs.

| Gene | Univariate Cox analysis | | | Multivariate Cox analysis | | |
| --- | --- | --- | --- | --- | --- | --- |
|  | Hazard Ratio | 95%CI | P Value | Hazard Ratio | 95%CI | P Value |
| CDKN2A | 3.03 | 1.66-5.51 | 0 | 0 | 0-0 | 0 |
| NBR1 | 0.16 | 0.07-0.37 | 0 | 0 | 0-0 | 0 |
| PRKAR1A | 0.3 | 0.16-0.55 | 0 | 53595730.08 | 14149677.39-203008323.31 | 0 |
| RHEB | 8.57 | 3.09-23.79 | 0 | 0 | 0-0 | 0 |
| PARP1 | 3.86 | 2.02-7.39 | 0 | 0.02 | 0-0.2 | 0.001 |
| ATG10 | 0.2 | 0.08-0.52 | 0.001 | 0 | 0-0 | 0 |
| BNIP1 | 6.13 | 2.12-17.74 | 0.001 | 2.56339E+14 | 18790647300056.3-3496940547816117 | 0 |
| EIF4EBP1 | 2.06 | 1.36-3.11 | 0.001 | 0.1 | 0.03-0.38 | 0.001 |
| DAPK1 | 0.64 | 0.48-0.85 | 0.002 | 3.33423E+22 | 1.62490427577777e+22-6.84167124173627e+22 | 0 |
| ERBB2 | 22.19 | 3.23-152.62 | 0.002 | 0 | 0-0 | 0 |
| ULK2 | 0.27 | 0.12-0.61 | 0.002 | 0 | 0-0 | 0 |
| HDAC1 | 2.6 | 1.38-4.91 | 0.003 | 1.42187E+29 | 3.78320855477815e+28-5.34388040219834e+29 | 0 |
| RPS6KB1 | 0.31 | 0.14-0.68 | 0.003 | 0 | 0-0 | 0 |
| SIRT1 | 0.38 | 0.2-0.72 | 0.003 | 0 | 0-0 | 0 |
| GAA | 0.31 | 0.14-0.68 | 0.004 | 0 | 0-0 | 0 |
| BIRC5 | 1.82 | 1.2-2.75 | 0.005 | 0 | 0-0 | 0 |
| RAC1 | 8.81 | 1.9-40.79 | 0.005 | 0 | 0-0 | 0 |
| CALCOCO2 | 0.19 | 0.06-0.62 | 0.006 | 0 | 0-0 | 0 |
| ATG2B | 0.23 | 0.08-0.67 | 0.007 | 1.03724E+63 | 1.58351483425255e+62-6.79412850479126e+63 | 0 |
| CAMKK2 | 11.43 | 1.95-67.05 | 0.007 | 8.4376E+88 | 4.95320080599753e+87-1.43731426391297e+90 | 0 |
| NFKB1 | 0.51 | 0.31-0.83 | 0.007 | 114891.98 | 36496.11-361686.91 | 0 |
| NRG1 | 2.39 | 1.27-4.48 | 0.007 | 0 | 0-0 | 0 |
| USP10 | 3.03 | 1.36-6.79 | 0.007 | 6.78 | 0.7-66.05 | 0.099 |
| MAPK8IP1 | 6.96 | 1.68-28.88 | 0.008 | 0 | 0-0 | 0 |
| ZFYVE1 | 0.19 | 0.06-0.66 | 0.008 | 252559361.2 | 34746570.61-1835756156.2 | 0 |
| CHMP4B | 2.76 | 1.29-5.9 | 0.009 | 1.52879E+32 | 3.03078939548002e+31-7.71155595017271e+32 | 0 |
| ITPR1 | 0.43 | 0.22-0.83 | 0.013 | 0 | 0-0 | 0 |
| MLST8 | 2.46 | 1.18-5.1 | 0.016 | 15406231529 | 3896701768.19-60910991919.23 | 0 |
| TSC1 | 0.37 | 0.17-0.83 | 0.016 | 0 | 0-0 | 0 |
| ULK3 | 0.42 | 0.2-0.86 | 0.017 | 6802562.52 | 1731437.44-26726265.52 | 0 |
| RAF1 | 0.4 | 0.19-0.85 | 0.018 | 2.83383E+13 | 5121538111734.88-156800128168670 | 0 |
| RGS19 | 1.97 | 1.12-3.46 | 0.018 | 1.85 | 0.29-11.92 | 0.517 |
| CAPNS1 | 0.41 | 0.2-0.87 | 0.019 | 0 | 0-0 | 0 |
| TSC2 | 0.34 | 0.14-0.84 | 0.019 | 2.19179E+53 | 4.16148906128662e+52-1.15438368630656e+54 | 0 |
| BID | 2.15 | 1.13-4.08 | 0.02 | 3.14261E+22 | 7.53606831751442e+21-1.31049379206554e+23 | 0 |
| CDKN1A | 0.67 | 0.48-0.94 | 0.02 | 4063260114 | 1362807113.71-12114761207.59 | 0 |
| EIF2AK2 | 0.24 | 0.07-0.81 | 0.021 | 5.58528E+13 | 1730648488786.85-1802525295667149 | 0 |
| ATG4C | 0.58 | 0.36-0.92 | 0.022 | 882.12 | 258.06-3015.33 | 0 |
| EDEM1 | 0.45 | 0.23-0.9 | 0.024 | 0 | 0-0 | 0 |
| CASP8 | 0.25 | 0.08-0.84 | 0.025 | 0 | 0-0 | 0 |
| SESN2 | 0.42 | 0.2-0.91 | 0.028 | 0 | 0-0 | 0 |
| ATG12 | 0.19 | 0.04-0.85 | 0.03 | 0 | 0-0 | 0 |
| SPNS1 | 2.84 | 1.08-7.5 | 0.035 | 0 | 0-0 | 0 |
| SQSTM1 | 0.28 | 0.09-0.92 | 0.036 | 0 | 0-0 | 0 |
| UVRAG | 0.58 | 0.34-0.97 | 0.04 | 0 | 0-0 | 0 |
| FOS | 0.81 | 0.67-0.99 | 0.042 | 0.05 | 0.03-0.08 | 0 |
| EIF4G1 | 2.86 | 1.03-7.98 | 0.044 | 1.34922E+22 | 1.68624502200977e+21-1.07955280093444e+23 | 0 |
| CTSB | 1.49 | 1.01-2.2 | 0.046 | 5.12541E+33 | 1.77903823915624e+33-1.47663168708715e+34 | 0 |
| PIK3C3 | 0.41 | 0.17-1.01 | 0.053 | #N/A | #N/A | #N/A |
| RAB5A | 0.3 | 0.09-1.03 | 0.055 | #N/A | #N/A | #N/A |
| HSPA5 | 2.05 | 0.95-4.42 | 0.067 | #N/A | #N/A | #N/A |
| ATG5 | 2.41 | 0.94-6.19 | 0.068 | #N/A | #N/A | #N/A |
| NCKAP1 | 1.73 | 0.94-3.17 | 0.076 | #N/A | #N/A | #N/A |
| CAPN2 | 0.49 | 0.22-1.08 | 0.078 | #N/A | #N/A | #N/A |
| MAP2K7 | 2.79 | 0.89-8.74 | 0.078 | #N/A | #N/A | #N/A |
| CHMP2B | 1.62 | 0.93-2.84 | 0.091 | #N/A | #N/A | #N/A |
| NAF1 | 0.7 | 0.47-1.06 | 0.092 | #N/A | #N/A | #N/A |
| GABARAP | 0.52 | 0.24-1.12 | 0.095 | #N/A | #N/A | #N/A |
| DAPK2 | 0.68 | 0.43-1.07 | 0.097 | #N/A | #N/A | #N/A |
| ITGA6 | 0.76 | 0.54-1.06 | 0.101 | #N/A | #N/A | #N/A |
| DNAJB9 | 0.68 | 0.43-1.09 | 0.107 | #N/A | #N/A | #N/A |
| ATG4A | 0.59 | 0.31-1.12 | 0.108 | #N/A | #N/A | #N/A |
| ATIC | 1.37 | 0.93-2 | 0.108 | #N/A | #N/A | #N/A |
| WDR45 | 0.57 | 0.29-1.13 | 0.109 | #N/A | #N/A | #N/A |
| EEF2 | 0.51 | 0.23-1.16 | 0.11 | #N/A | #N/A | #N/A |
| NRG2 | 3.2 | 0.76-13.4 | 0.111 | #N/A | #N/A | #N/A |
| PELP1 | 2.23 | 0.83-6 | 0.112 | #N/A | #N/A | #N/A |
| ULK1 | 1.87 | 0.86-4.06 | 0.113 | #N/A | #N/A | #N/A |
| FAS | 0.62 | 0.34-1.12 | 0.115 | #N/A | #N/A | #N/A |
| TP73 | 2.2 | 0.81-6.01 | 0.123 | #N/A | #N/A | #N/A |
| NKX2_3 | 0.82 | 0.64-1.06 | 0.138 | #N/A | #N/A | #N/A |
| MAP1LC3B | 0.65 | 0.37-1.15 | 0.139 | #N/A | #N/A | #N/A |
| MTOR | 2.8 | 0.71-10.96 | 0.139 | #N/A | #N/A | #N/A |
| RELA | 0.45 | 0.15-1.31 | 0.141 | #N/A | #N/A | #N/A |
| SIRT2 | 0.48 | 0.18-1.29 | 0.146 | #N/A | #N/A | #N/A |
| PRKAB1 | 1.85 | 0.8-4.25 | 0.148 | #N/A | #N/A | #N/A |
| TBK1 | 1.38 | 0.88-2.15 | 0.158 | #N/A | #N/A | #N/A |
| RPTOR | 0.45 | 0.15-1.37 | 0.161 | #N/A | #N/A | #N/A |
| NLRC4 | 2.53 | 0.68-9.41 | 0.165 | #N/A | #N/A | #N/A |
| MAPK1 | 2.49 | 0.67-9.28 | 0.176 | #N/A | #N/A | #N/A |
| VAMP3 | 0.71 | 0.44-1.16 | 0.176 | #N/A | #N/A | #N/A |
| VEGFA | 1.35 | 0.87-2.08 | 0.176 | #N/A | #N/A | #N/A |
| TP53 | 1.61 | 0.8-3.26 | 0.183 | #N/A | #N/A | #N/A |
| GABARAPL2 | 0.68 | 0.38-1.21 | 0.19 | #N/A | #N/A | #N/A |
| CAPN1 | 0.54 | 0.22-1.36 | 0.192 | #N/A | #N/A | #N/A |
| SAR1A | 0.57 | 0.25-1.32 | 0.192 | #N/A | #N/A | #N/A |
| CASP1 | 0.8 | 0.57-1.12 | 0.196 | #N/A | #N/A | #N/A |
| CASP3 | 1.55 | 0.79-3.06 | 0.204 | #N/A | #N/A | #N/A |
| BAK1 | 1.51 | 0.79-2.9 | 0.21 | #N/A | #N/A | #N/A |
| APOL1 | 1.42 | 0.81-2.51 | 0.219 | #N/A | #N/A | #N/A |
| DLC1 | 1.6 | 0.74-3.47 | 0.23 | #N/A | #N/A | #N/A |
| BCL2L1 | 1.81 | 0.67-4.87 | 0.239 | #N/A | #N/A | #N/A |
| ITGA3 | 1.42 | 0.79-2.56 | 0.244 | #N/A | #N/A | #N/A |
| MYC | 0.86 | 0.66-1.11 | 0.247 | #N/A | #N/A | #N/A |
| GRID1 | 2.16 | 0.57-8.19 | 0.256 | #N/A | #N/A | #N/A |
| ITGB4 | 1.85 | 0.62-5.54 | 0.273 | #N/A | #N/A | #N/A |
| IKBKB | 0.63 | 0.27-1.45 | 0.275 | #N/A | #N/A | #N/A |
| WIPI2 | 2.01 | 0.56-7.29 | 0.287 | #N/A | #N/A | #N/A |
| BIRC6 | 0.66 | 0.31-1.43 | 0.291 | #N/A | #N/A | #N/A |
| BAG1 | 0.58 | 0.22-1.59 | 0.292 | #N/A | #N/A | #N/A |
| RB1 | 0.64 | 0.28-1.46 | 0.292 | #N/A | #N/A | #N/A |
| TM9SF1 | 0.56 | 0.19-1.66 | 0.293 | #N/A | #N/A | #N/A |
| RB1CC1 | 0.59 | 0.22-1.57 | 0.294 | #N/A | #N/A | #N/A |
| LAMP2 | 0.75 | 0.45-1.28 | 0.296 | #N/A | #N/A | #N/A |
| WDFY3 | 0.71 | 0.37-1.35 | 0.296 | #N/A | #N/A | #N/A |
| ARSB | 1.96 | 0.55-6.98 | 0.299 | #N/A | #N/A | #N/A |
| ATF4 | 1.58 | 0.66-3.8 | 0.304 | #N/A | #N/A | #N/A |
| PPP1R15A | 0.8 | 0.52-1.23 | 0.304 | #N/A | #N/A | #N/A |
| TP53INP2 | 1.32 | 0.76-2.3 | 0.32 | #N/A | #N/A | #N/A |
| KLHL24 | 0.6 | 0.22-1.68 | 0.333 | #N/A | #N/A | #N/A |
| IFNG | 0.82 | 0.55-1.22 | 0.335 | #N/A | #N/A | #N/A |
| DDIT3 | 1.3 | 0.76-2.24 | 0.339 | #N/A | #N/A | #N/A |
| TNFSF10 | 0.88 | 0.67-1.16 | 0.368 | #N/A | #N/A | #N/A |
| BCL2 | 1.41 | 0.66-3.02 | 0.374 | #N/A | #N/A | #N/A |
| NFE2L2 | 0.59 | 0.19-1.89 | 0.378 | #N/A | #N/A | #N/A |
| PEX14 | 1.78 | 0.47-6.73 | 0.393 | #N/A | #N/A | #N/A |
| ERN1 | 0.71 | 0.32-1.57 | 0.398 | #N/A | #N/A | #N/A |
| GRID2 | 1.69 | 0.49-5.8 | 0.403 | #N/A | #N/A | #N/A |
| TMEM74 | 0.73 | 0.35-1.52 | 0.403 | #N/A | #N/A | #N/A |
| CANX | 1.58 | 0.53-4.71 | 0.415 | #N/A | #N/A | #N/A |
| CX3CL1 | 0.8 | 0.47-1.37 | 0.418 | #N/A | #N/A | #N/A |
| HSPB8 | 1.16 | 0.81-1.66 | 0.419 | #N/A | #N/A | #N/A |
| SPHK1 | 1.3 | 0.68-2.48 | 0.434 | #N/A | #N/A | #N/A |
| FADD | 0.75 | 0.37-1.54 | 0.439 | #N/A | #N/A | #N/A |
| ATG7 | 2.04 | 0.32-12.97 | 0.45 | #N/A | #N/A | #N/A |
| ATF6 | 1.37 | 0.6-3.12 | 0.454 | #N/A | #N/A | #N/A |
| TUSC1 | 0.87 | 0.61-1.25 | 0.454 | #N/A | #N/A | #N/A |
| ATG9A | 0.72 | 0.29-1.79 | 0.485 | #N/A | #N/A | #N/A |
| CDKN1B | 0.85 | 0.53-1.36 | 0.495 | #N/A | #N/A | #N/A |
| HGS | 1.4 | 0.53-3.68 | 0.497 | #N/A | #N/A | #N/A |
| DNAJB1 | 1.12 | 0.81-1.54 | 0.499 | #N/A | #N/A | #N/A |
| DIRAS3 | 0.86 | 0.56-1.33 | 0.507 | #N/A | #N/A | #N/A |
| MAPK9 | 0.71 | 0.26-1.95 | 0.511 | #N/A | #N/A | #N/A |
| PEA15 | 1.13 | 0.79-1.61 | 0.512 | #N/A | #N/A | #N/A |
| RAB7A | 2 | 0.25-16.07 | 0.515 | #N/A | #N/A | #N/A |
| LAMP1 | 1.24 | 0.63-2.44 | 0.526 | #N/A | #N/A | #N/A |
| PEX3 | 1.35 | 0.52-3.5 | 0.537 | #N/A | #N/A | #N/A |
| STK11 | 0.73 | 0.27-1.99 | 0.54 | #N/A | #N/A | #N/A |
| MAPK8 | 0.71 | 0.24-2.14 | 0.541 | #N/A | #N/A | #N/A |
| RAB11A | 1.17 | 0.71-1.92 | 0.547 | #N/A | #N/A | #N/A |
| CCR2 | 0.85 | 0.5-1.44 | 0.553 | #N/A | #N/A | #N/A |
| ST13 | 0.87 | 0.55-1.38 | 0.557 | #N/A | #N/A | #N/A |
| RAB33B | 0.82 | 0.41-1.67 | 0.589 | #N/A | #N/A | #N/A |
| ARSA | 0.76 | 0.27-2.12 | 0.594 | #N/A | #N/A | #N/A |
| ARNT | 0.72 | 0.22-2.41 | 0.595 | #N/A | #N/A | #N/A |
| PARK2 | 1.45 | 0.34-6.19 | 0.619 | #N/A | #N/A | #N/A |
| ATG3 | 1.29 | 0.47-3.54 | 0.626 | #N/A | #N/A | #N/A |
| BAG3 | 0.88 | 0.53-1.47 | 0.629 | #N/A | #N/A | #N/A |
| KIF5B | 0.79 | 0.29-2.15 | 0.651 | #N/A | #N/A | #N/A |
| PINK1 | 0.83 | 0.37-1.86 | 0.656 | #N/A | #N/A | #N/A |
| PIK3R4 | 0.61 | 0.06-6.12 | 0.673 | #N/A | #N/A | #N/A |
| BAX | 0.8 | 0.27-2.38 | 0.692 | #N/A | #N/A | #N/A |
| MAPK3 | 1.29 | 0.36-4.61 | 0.697 | #N/A | #N/A | #N/A |
| ITGB1 | 0.86 | 0.4-1.87 | 0.702 | #N/A | #N/A | #N/A |
| MAP1LC3A | 1.19 | 0.48-2.97 | 0.704 | #N/A | #N/A | #N/A |
| GOPC | 0.84 | 0.33-2.14 | 0.709 | #N/A | #N/A | #N/A |
| EGFR | 1.16 | 0.5-2.68 | 0.729 | #N/A | #N/A | #N/A |
| HDAC6 | 1.29 | 0.29-5.64 | 0.736 | #N/A | #N/A | #N/A |
| CCL2 | 0.97 | 0.8-1.18 | 0.753 | #N/A | #N/A | #N/A |
| MTMR14 | 0.78 | 0.17-3.64 | 0.756 | #N/A | #N/A | #N/A |
| RAB1A | 0.87 | 0.35-2.15 | 0.761 | #N/A | #N/A | #N/A |
| FOXO1 | 0.87 | 0.34-2.23 | 0.777 | #N/A | #N/A | #N/A |
| EIF2S1 | 1.14 | 0.45-2.89 | 0.778 | #N/A | #N/A | #N/A |
| HIF1A | 0.93 | 0.53-1.62 | 0.798 | #N/A | #N/A | #N/A |
| PRKCD | 0.95 | 0.61-1.46 | 0.8 | #N/A | #N/A | #N/A |
| GNAI3 | 1.09 | 0.54-2.22 | 0.804 | #N/A | #N/A | #N/A |
| AMBRA1 | 1.33 | 0.14-12.95 | 0.806 | #N/A | #N/A | #N/A |
| NPC1 | 0.87 | 0.19-3.95 | 0.858 | #N/A | #N/A | #N/A |
| PTK6 | 1.07 | 0.37-3.06 | 0.9 | #N/A | #N/A | #N/A |
| DRAM1 | 1.02 | 0.75-1.38 | 0.918 | #N/A | #N/A | #N/A |
| CXCR4 | 0.99 | 0.78-1.25 | 0.92 | #N/A | #N/A | #N/A |
| PRKCQ | 0.98 | 0.61-1.58 | 0.928 | #N/A | #N/A | #N/A |
| BNIP3L | 0.98 | 0.55-1.73 | 0.938 | #N/A | #N/A | #N/A |
| TP63 | 1.03 | 0.35-3.02 | 0.955 | #N/A | #N/A | #N/A |
| SH3GLB1 | 1.01 | 0.61-1.69 | 0.964 | #N/A | #N/A | #N/A |
| FOXO3 | 1.01 | 0.43-2.35 | 0.98 | #N/A | #N/A | #N/A |
