# Supplementary material for: The prognostic value of autophagy related genes with potential protective function in Ewing sarcoma
Source: BMC Bioinformatics. 2022 Jul 28;23:306. doi: 10.1186/s12859-022-04849-x (PMC9335970; doi:10.1186/s12859-022-04849-x)
Supplement: Supplementary file 2 — Additional file 2. DEGs identified by “limma” between high and low score groups. [file 12859_2022_4849_MOESM2_ESM.docx]

**Additional file 2** DEGs identified by “limma” between high and low score groups

|  | symbol | logFC | AveExpr | t | P.Value | adj.P.Val | B |
| --- | --- | --- | --- | --- | --- | --- | --- |
| 1 | DAPK1 | 1.239288075 | 7.511087324 | 7.973949154 | 3.37997E-11 | 1.61299E-07 | 15.2300249 |
| 2 | PHYHD1 | 1.055278029 | 5.583341497 | 7.96336813 | 3.52901E-11 | 1.61299E-07 | 15.18899687 |
| 3 | NTNG1 | 1.113379502 | 5.561466472 | 7.751275992 | 8.38174E-11 | 1.99251E-07 | 14.36625093 |
| 4 | DFNB59 | 1.439501518 | 5.219903661 | 7.506168848 | 2.27718E-10 | 2.7842E-07 | 13.4151864 |
| 5 | DAPK1-IT1 | 1.903051331 | 7.62494456 | 7.505231654 | 2.28589E-10 | 2.7842E-07 | 13.41155066 |
| 6 | KDSR | 1.370785377 | 7.133494441 | 7.459393893 | 2.75541E-10 | 2.7842E-07 | 13.2337425 |
| 7 | ENPP3 | 1.252908115 | 5.184069151 | 7.446992011 | 2.89824E-10 | 2.7842E-07 | 13.18564018 |
| 8 | TMCO6 | 1.007343702 | 5.783087236 | 7.404772495 | 3.4422E-10 | 3.12013E-07 | 13.02190691 |
| 9 | EWSAT1 | 1.356523297 | 5.600434306 | 6.637410536 | 7.73412E-09 | 2.48063E-06 | 10.05863548 |
| 10 | EZH2 | 1.394796812 | 8.639248213 | 6.520572909 | 1.23765E-08 | 3.26614E-06 | 9.610998248 |
| 11 | CDC37L1 | 1.447887306 | 6.077185945 | 6.490864167 | 1.39451E-08 | 3.54547E-06 | 9.497389947 |
| 12 | ITGB2-AS1 | 1.875497589 | 7.076359134 | 6.48241856 | 1.44261E-08 | 3.62356E-06 | 9.465110073 |
| 13 | FBXO15 | 1.034745162 | 5.317580601 | 6.368310126 | 2.27918E-08 | 4.94962E-06 | 9.029740639 |
| 14 | CDH8 | 1.562808724 | 5.199285832 | 6.2939653 | 3.0679E-08 | 6.10209E-06 | 8.746899908 |
| 15 | PAXIP1-AS1 | 1.206015519 | 6.061644492 | 6.219750217 | 4.12459E-08 | 7.38622E-06 | 8.465247193 |
| 16 | RBM4 | 1.1520951 | 5.965013719 | 6.12446273 | 6.02493E-08 | 9.23586E-06 | 8.104714633 |
| 17 | JAK1 | 1.155135414 | 8.505169797 | 6.093903257 | 6.80166E-08 | 1.02754E-05 | 7.989363702 |
| 18 | TRPM4 | 1.249658673 | 7.82310538 | 6.017301719 | 9.21212E-08 | 1.26273E-05 | 7.700839871 |
| 19 | DLG2 | 1.636768301 | 6.329998318 | 5.95905286 | 1.15952E-07 | 1.51086E-05 | 7.482060907 |
| 20 | DCC | 1.161150506 | 4.758157024 | 5.914651635 | 1.3813E-07 | 1.69082E-05 | 7.31566824 |
| 21 | GIMAP2 | 1.436540757 | 6.487581303 | 5.903838744 | 1.44137E-07 | 1.70136E-05 | 7.275197729 |
| 22 | OGDHL | 1.198021803 | 6.050229267 | 5.8829092 | 1.56508E-07 | 1.74663E-05 | 7.19691992 |
| 23 | ADRB1 | 1.039994009 | 5.447595823 | 5.814192142 | 2.04987E-07 | 2.06234E-05 | 6.940458339 |
| 24 | SHTN1 | 1.293887098 | 7.215580651 | 5.780657523 | 2.33769E-07 | 2.2459E-05 | 6.81561452 |
| 25 | MOK | 1.05039765 | 6.206516678 | 5.733577807 | 2.81024E-07 | 2.5145E-05 | 6.640702005 |
| 26 | OPLAH | 1.079846788 | 6.310829163 | 5.694317177 | 3.27554E-07 | 2.79505E-05 | 6.495167332 |
| 27 | MYL5 | 1.223274778 | 5.756252404 | 5.657707714 | 3.77759E-07 | 3.12521E-05 | 6.359736475 |
| 28 | PAPPA | 1.209631114 | 6.411451144 | 5.601613486 | 4.69773E-07 | 3.61396E-05 | 6.152757184 |
| 29 | DCDC2 | 1.62975067 | 5.394304258 | 5.56431276 | 5.42862E-07 | 3.95227E-05 | 6.015489815 |
| 30 | C15orf52 | 1.205899177 | 6.118675312 | 5.528329256 | 6.23958E-07 | 4.2956E-05 | 5.883355124 |
| 31 | CYP26B1 | 1.255489977 | 6.343724243 | 5.524656948 | 6.32877E-07 | 4.32598E-05 | 5.869886082 |
| 32 | HOOK1 | 1.669758302 | 7.499324436 | 5.497666044 | 7.02404E-07 | 4.57616E-05 | 5.770982935 |
| 33 | ST6GALNAC2 | 1.031064487 | 4.7908676 | 5.477563852 | 7.59027E-07 | 4.81255E-05 | 5.6974288 |
| 34 | DCLRE1A | 1.092583003 | 5.582306009 | 5.464937371 | 7.96868E-07 | 4.94201E-05 | 5.65127548 |
| 35 | SCNN1G | 1.106107874 | 5.639542309 | 5.409411487 | 9.86557E-07 | 5.76235E-05 | 5.44875195 |
| 36 | CLEC11A | 1.321674856 | 8.015852343 | 5.381838788 | 1.09663E-06 | 6.14584E-05 | 5.348455066 |
| 37 | QRFPR | 2.093350041 | 4.873702882 | 5.357134311 | 1.20547E-06 | 6.52768E-05 | 5.258747489 |
| 38 | PAQR5 | 1.190171306 | 4.889956988 | 5.156582843 | 2.58466E-06 | 0.000106915 | 4.536230316 |
| 39 | PLPP6 | 1.315860832 | 6.794208438 | 5.152074771 | 2.62905E-06 | 0.000107992 | 4.520111725 |
| 40 | JAKMIP2 | 1.217252383 | 7.384544299 | 5.071025146 | 3.56752E-06 | 0.000132577 | 4.231286425 |
| 41 | DNAL4 | 1.188624593 | 7.22483778 | 5.064571411 | 3.65502E-06 | 0.000135106 | 4.208368218 |
| 42 | PRKCB | 1.239568673 | 7.402086757 | 5.048409906 | 3.88347E-06 | 0.00013935 | 4.151029011 |
| 43 | NCKAP5 | 1.073321919 | 4.442284324 | 5.033516263 | 4.10636E-06 | 0.000144367 | 4.098255264 |
| 44 | LIPI | 2.390303205 | 7.121831891 | 5.02718204 | 4.20492E-06 | 0.000146841 | 4.075830417 |
| 45 | CCND1 | 1.028573935 | 10.06911005 | 4.94451672 | 5.7244E-06 | 0.000181647 | 3.784268918 |
| 46 | LOC100288152 | 1.225657168 | 6.20518974 | 4.942583159 | 5.76572E-06 | 0.000182019 | 3.777473975 |
| 47 | SLFN11 | 1.209367214 | 7.741955134 | 4.940636125 | 5.80762E-06 | 0.00018262 | 3.770632845 |
| 48 | ASIP | 1.024018079 | 4.29059866 | 4.836800672 | 8.53121E-06 | 0.000235963 | 3.407512011 |
| 49 | SORD | 1.016207628 | 7.088525562 | 4.808866247 | 9.45587E-06 | 0.000254698 | 3.310411421 |
| 50 | GLCE | 1.272115792 | 7.493203074 | 4.80626902 | 9.54666E-06 | 0.000256605 | 3.301396364 |
| 51 | PARP11 | 1.095381684 | 5.643872527 | 4.752983025 | 1.16089E-05 | 0.000288466 | 3.116932131 |
| 52 | PTPN22 | 1.130975866 | 3.998329631 | 4.743294207 | 1.2028E-05 | 0.000296758 | 3.08349371 |
| 53 | ZDHHC21 | 1.005739714 | 5.645656847 | 4.731098114 | 1.25767E-05 | 0.000305782 | 3.041447285 |
| 54 | CHRDL1 | 1.785228107 | 6.621012484 | 4.643031019 | 1.73308E-05 | 0.00038033 | 2.73935473 |
| 55 | LOC101928047 | 1.374528742 | 4.135315905 | 4.574369386 | 2.22135E-05 | 0.000451201 | 2.505731625 |
| 56 | AMER2 | 1.336267611 | 9.734074565 | 4.560203482 | 2.3376E-05 | 0.000463088 | 2.457744806 |
| 57 | CAMK1G | 1.240991237 | 5.437470485 | 4.527986547 | 2.62447E-05 | 0.000496056 | 2.348886439 |
| 58 | SLC38A5 | 1.256098707 | 6.485841253 | 4.497480091 | 2.92752E-05 | 0.000535846 | 2.246165256 |
| 59 | PGLYRP2 | 1.397570565 | 6.886742727 | 4.484745858 | 3.06385E-05 | 0.000550939 | 2.203390679 |
| 60 | CYP4F22 | 1.556511196 | 7.821794745 | 4.461978668 | 3.32313E-05 | 0.000578303 | 2.127069419 |
| 61 | ADRA1D | 1.069459963 | 6.001433764 | 4.454607083 | 3.41156E-05 | 0.00058936 | 2.102400706 |
| 62 | CCDC171 | 1.270851673 | 5.711219264 | 4.439924735 | 3.59456E-05 | 0.000606797 | 2.053329412 |
| 63 | TTLL6 | 1.225882511 | 5.414185903 | 4.40087192 | 4.12894E-05 | 0.000671503 | 1.923216467 |
| 64 | TMEM158 | 1.118561854 | 6.388105011 | 4.398846277 | 4.15866E-05 | 0.000673658 | 1.916483955 |
| 65 | ABCA5 | 1.528030586 | 6.618853591 | 4.313688661 | 5.61475E-05 | 0.000830575 | 1.634936688 |
| 66 | ZMAT4 | 1.244030093 | 6.195562707 | 4.310328908 | 5.68133E-05 | 0.000837655 | 1.623889001 |
| 67 | ALDH7A1 | 1.022511923 | 7.309285324 | 4.279501739 | 6.32901E-05 | 0.000900029 | 1.52273905 |
| 68 | CEACAM6 | 2.147732262 | 6.187336281 | 4.265258833 | 6.65188E-05 | 0.000931353 | 1.476138531 |
| 69 | ITM2A | 1.189271194 | 11.21468636 | 4.263428436 | 6.69451E-05 | 0.000934811 | 1.470155911 |
| 70 | C1orf226 | 1.412555916 | 5.397721479 | 4.230796674 | 7.50045E-05 | 0.001011444 | 1.363736065 |
| 71 | TMEM71 | 1.288581731 | 5.953820821 | 4.150038923 | 9.91922E-05 | 0.001226547 | 1.102322419 |
| 72 | SPHKAP | 1.862953763 | 5.53158303 | 4.143537431 | 0.000101438 | 0.001239612 | 1.081400203 |
| 73 | LECT1 | 1.405227083 | 7.776288945 | 4.092020405 | 0.000121054 | 0.001392792 | 0.916276226 |
| 74 | GSTM5 | 1.039911825 | 6.548877376 | 4.088826249 | 0.000122384 | 0.001401097 | 0.906077184 |
| 75 | SYNDIG1 | 1.062349072 | 5.002955414 | 4.045799853 | 0.000141729 | 0.001544574 | 0.769141669 |
| 76 | ADGRG2 | 2.441405547 | 8.561901246 | 4.02880655 | 0.000150154 | 0.00159899 | 0.715291147 |
| 77 | MSC | 1.179398837 | 6.106189496 | 4.014290405 | 0.000157732 | 0.00164749 | 0.669395713 |
| 78 | STEAP3 | 1.410508196 | 5.723344516 | 3.975902521 | 0.000179586 | 0.001808698 | 0.548496565 |
| 79 | TRIM35 | 1.012272704 | 7.022045017 | 3.958631181 | 0.000190343 | 0.001886057 | 0.494327058 |
| 80 | ARX | 1.025101676 | 6.410328752 | 3.957738029 | 0.000190915 | 0.001890834 | 0.491529617 |
| 81 | ENPP1 | 1.364778509 | 5.996018627 | 3.956847754 | 0.000191488 | 0.001893806 | 0.488741565 |
| 82 | LOXHD1 | 1.657565636 | 7.081511023 | 3.933800873 | 0.000206899 | 0.001996958 | 0.416696939 |
| 83 | CRIP1 | 1.257199403 | 8.248773826 | 3.907115979 | 0.000226232 | 0.002126463 | 0.333596465 |
| 84 | CSPG5 | 1.047079765 | 6.998927491 | 3.90690322 | 0.000226393 | 0.002127016 | 0.332935277 |
| 85 | POU4F2 | 1.951201637 | 5.431988946 | 3.891627876 | 0.000238237 | 0.002194774 | 0.285521452 |
| 86 | RBM11 | 1.546703224 | 7.918857341 | 3.862140299 | 0.000262805 | 0.002353502 | 0.194313939 |
| 87 | RGS2 | -1.371861295 | 7.17075234 | -3.847620548 | 0.000275777 | 0.00243044 | 0.149559337 |
| 88 | HLF | 1.03786593 | 4.674482204 | 3.826981163 | 0.000295279 | 0.00254064 | 0.086120817 |
| 89 | XG | 1.268818702 | 6.632264455 | 3.815814329 | 0.000306374 | 0.002607054 | 0.051885732 |
| 90 | SLC38A4 | 1.560363973 | 4.153328479 | 3.79536366 | 0.000327737 | 0.002724346 | -0.010650346 |
| 91 | PPP1R1A | 1.323145208 | 7.859892354 | 3.763964614 | 0.000363335 | 0.00293143 | -0.106256425 |
| 92 | SIAH3 | 1.487708647 | 5.26718817 | 3.704682602 | 0.000440872 | 0.00336185 | -0.285393901 |
| 93 | OLFM3 | 1.564999133 | 5.512462946 | 3.695732168 | 0.000453871 | 0.003423412 | -0.312282855 |
| 94 | SAMD11 | 1.021398357 | 6.368068834 | 3.691265273 | 0.000460495 | 0.003459606 | -0.325686761 |
| 95 | HSD11B1 | 1.385577568 | 5.417938117 | 3.678988633 | 0.000479179 | 0.00356528 | -0.362471965 |
| 96 | NKX2-2 | 1.067846696 | 9.454992204 | 3.660749859 | 0.000508276 | 0.003725931 | -0.416976224 |
| 97 | NXN | -1.047319359 | 7.327443513 | -3.652525583 | 0.000521942 | 0.003788803 | -0.441496223 |
| 98 | TSPAN8 | 2.650364294 | 8.96114596 | 3.625246788 | 0.00056982 | 0.004016091 | -0.522569566 |
| 99 | CSTA | -1.147204084 | 5.833505743 | -3.563391227 | 0.000694352 | 0.004594051 | -0.704931534 |
| 100 | ZBTB16 | 1.015848516 | 5.704475713 | 3.495147268 | 0.000861648 | 0.005352691 | -0.903710688 |
| 101 | HMCN1 | 1.051580654 | 9.601340472 | 3.484439044 | 0.000891143 | 0.005473941 | -0.934667518 |
| 102 | SLC7A5 | 1.111808776 | 7.428077065 | 3.480578811 | 0.000902009 | 0.005524409 | -0.945811525 |
| 103 | SLC17A8 | 2.07408765 | 5.481219636 | 3.474035952 | 0.000920714 | 0.005599486 | -0.964680899 |
| 104 | SULF2 | -1.081009572 | 5.518908992 | -3.4727768 | 0.000924355 | 0.005613445 | -0.968309498 |
| 105 | C8orf4 | -1.408570877 | 5.087641182 | -3.453321133 | 0.000982387 | 0.005828344 | -1.024263228 |
| 106 | PTGDS | 1.064869545 | 7.007327208 | 3.426790065 | 0.001067104 | 0.006162599 | -1.100221045 |
| 107 | PARM1 | 1.128784263 | 6.890203062 | 3.405601018 | 0.001139683 | 0.006446041 | -1.160597205 |
| 108 | HIST1H2AC | 1.201020992 | 8.686588823 | 3.361964707 | 0.001304111 | 0.007095019 | -1.284122273 |
| 109 | MFAP2 | -1.009469921 | 7.196242568 | -3.343832182 | 0.001378822 | 0.007383943 | -1.335127049 |
| 110 | TSTD1 | 1.070128345 | 6.794333402 | 3.30674237 | 0.0015444 | 0.008031341 | -1.438856907 |
| 111 | APELA | 1.602649161 | 5.192223305 | 3.279764569 | 0.001676418 | 0.008514001 | -1.513795903 |
| 112 | NPW | 1.120605782 | 5.825442618 | 3.273495364 | 0.001708583 | 0.008639473 | -1.531148514 |
| 113 | PRSS35 | 1.519860294 | 8.066978214 | 3.218218281 | 0.002018411 | 0.009742959 | -1.683129469 |
| 114 | NKX2-3 | 1.314518754 | 3.577491899 | 3.215891202 | 0.002032547 | 0.009799847 | -1.68948713 |
| 115 | NPY1R | 1.548831851 | 9.101649054 | 3.206868562 | 0.002088237 | 0.009989805 | -1.714106153 |
| 116 | TRG-AS1 | 1.491340742 | 6.172030741 | 3.19414139 | 0.002169231 | 0.010273543 | -1.7487489 |
| 117 | REEP1 | 1.076548249 | 7.220257184 | 3.178631838 | 0.002271916 | 0.010612794 | -1.790831127 |
| 118 | TNFAIP6 | 1.213390785 | 9.073811502 | 3.145595747 | 0.002506013 | 0.011424743 | -1.879974466 |
| 119 | PADI2 | 1.158663244 | 5.556084565 | 3.142302875 | 0.002530545 | 0.01150388 | -1.888822804 |
| 120 | BEX1 | 1.195130829 | 9.679514224 | 3.097357439 | 0.002888655 | 0.012641566 | -2.008920104 |
| 121 | SYT1 | 1.249410422 | 5.973289088 | 3.088286721 | 0.002966445 | 0.012846788 | -2.033003966 |
| 122 | SYT4 | 1.691904475 | 6.498892693 | 3.010246624 | 0.00372118 | 0.015208105 | -2.238049765 |
| 123 | KL | 1.124094158 | 4.22353186 | 2.992620776 | 0.003914746 | 0.015746599 | -2.283819463 |
| 124 | PKP1 | 1.161615921 | 6.643307829 | 2.984161219 | 0.004010935 | 0.016022219 | -2.305715189 |
| 125 | EGR2 | 1.26032205 | 8.143488125 | 2.944123204 | 0.004496687 | 0.017366974 | -2.40871164 |
| 126 | BDP1 | 1.019102211 | 8.415510131 | 2.868343287 | 0.005568421 | 0.020327515 | -2.600757616 |
| 127 | STEAP1 | 1.012560532 | 9.699234273 | 2.854632458 | 0.00578589 | 0.020916288 | -2.635094844 |
| 128 | ZFPM2 | 1.038139383 | 4.864568702 | 2.779619411 | 0.007120716 | 0.024370177 | -2.820702043 |
| 129 | NR0B1 | 1.002296723 | 6.879392838 | 2.733800295 | 0.008069662 | 0.026789221 | -2.932175986 |
| 130 | LINC01405 | 1.085390806 | 4.924762332 | 2.723947439 | 0.008288294 | 0.027320608 | -2.955956967 |
| 131 | PRAME | -1.35886981 | 6.284112395 | -2.710324075 | 0.00859953 | 0.028166315 | -2.988726959 |
| 132 | PCDH8 | 1.406236642 | 7.900994297 | 2.692794411 | 0.00901573 | 0.029145595 | -3.030702154 |
| 133 | GDF10 | 1.515722191 | 6.109584242 | 2.617874575 | 0.011009915 | 0.033844693 | -3.207653847 |
| 134 | SYNPR | 1.596600342 | 6.830428678 | 2.608593236 | 0.011283082 | 0.034506335 | -3.229296974 |
| 135 | TUBB2B | -1.07530962 | 4.482077182 | -2.46687304 | 0.016291809 | 0.045565406 | -3.552012305 |
| 136 | KRT40 | 1.073290114 | 3.743124026 | 2.449138791 | 0.017042576 | 0.04711042 | -3.591356054 |
| 137 | G0S2 | 1.391759982 | 7.285994971 | 2.44171889 | 0.017365805 | 0.047693624 | -3.607747737 |
