# Supplementary material for: The prognostic value of autophagy related genes with potential protective function in Ewing sarcoma
Source: BMC Bioinformatics. 2022 Jul 28;23:306. doi: 10.1186/s12859-022-04849-x (PMC9335970; doi:10.1186/s12859-022-04849-x)
Supplement: Supplementary file 3 — Additional file 3. Box plots and density plots for gene expression profile of samples. Box plots for gene expression in GSE17679 (A) and GSE63155 (B): x-axis was the samples, y-axis was the expression of gene. Density plots for gene expression in GSE17679 (C) and GSE63155 (D): x-axis referred to the gene intensity, y-axis referred to the expression density of genes converted by log2. [file 12859_2022_4849_MOESM3_ESM.docx]

**
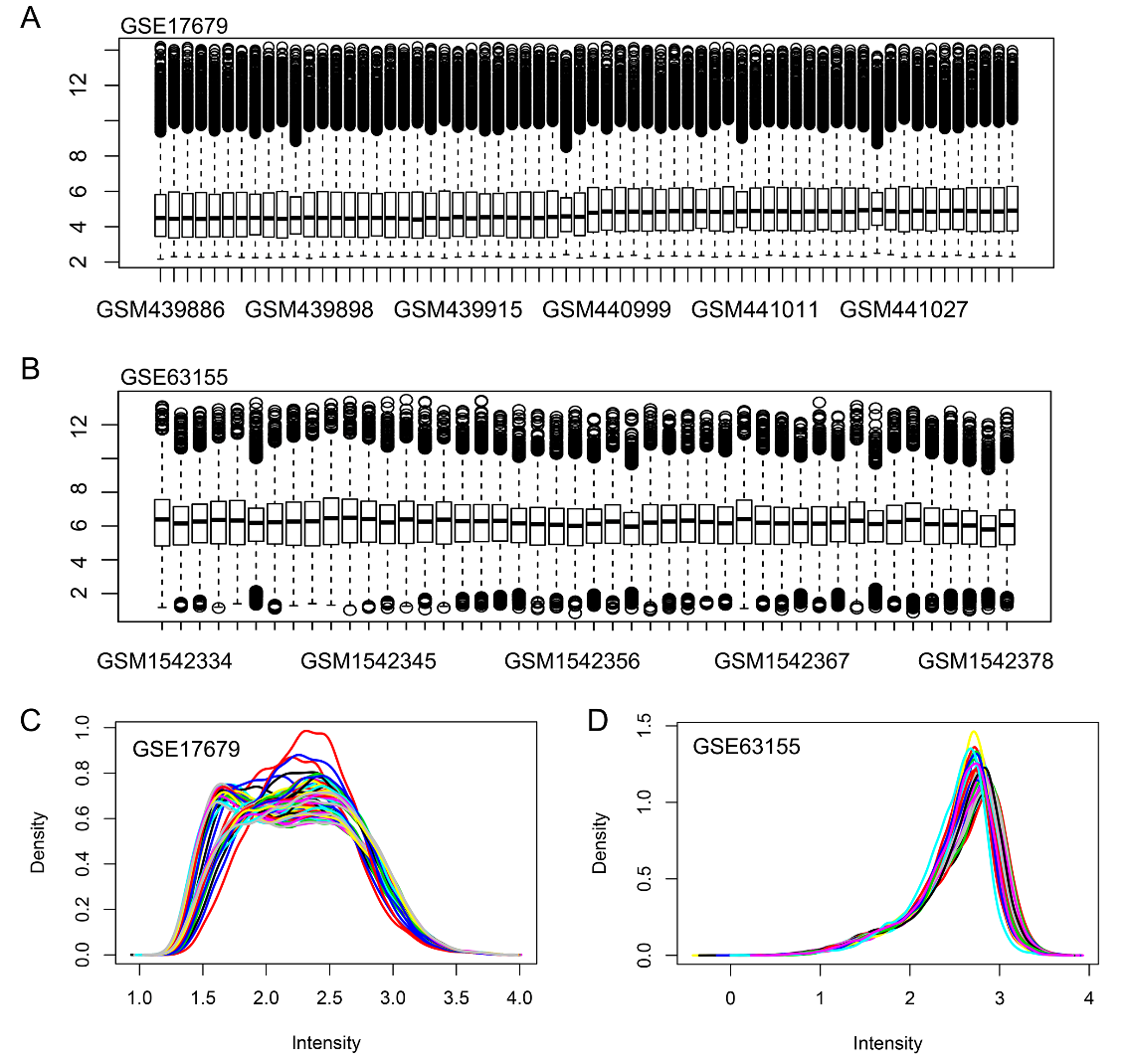
**

**Additional file 3** Box plots and density plots for gene expression profile of samples. Box plots for gene expression in GSE17679 (A) and GSE63155 (B): x-axis was the samples, y-axis was the expression of gene. Density plots for gene expression in GSE17679 (C) and GSE63155 (D): x-axis referred to the gene intensity, y-axis referred to the expression density of genes converted by log2.
