# Supplementary figures and images for: The prognostic value of autophagy related genes with potential protective function in Ewing sarcoma
Source: BMC Bioinformatics. 2022 Jul 28;23:306. doi: 10.1186/s12859-022-04849-x (PMC9335970; doi:10.1186/s12859-022-04849-x)

**
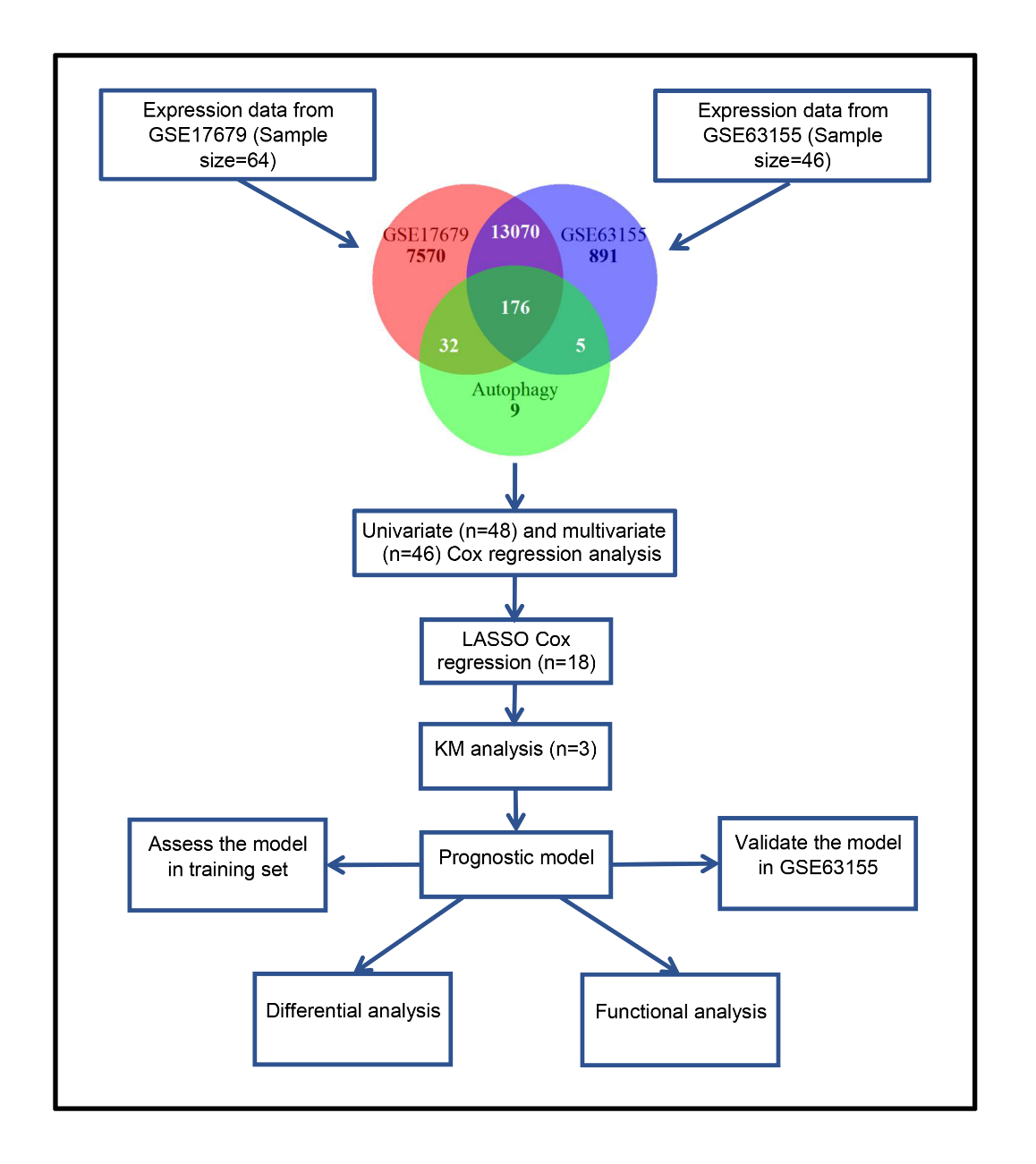
**

**Additional file 4** Flow chart of this study.

Supplement: Supplementary file 4 — Additional file 4. Flow chart of this study. [file 12859_2022_4849_MOESM4_ESM.docx]
